# DevKidCC allows for robust classification and direct comparisons of kidney organoid datasets

**DOI:** 10.1186/s13073-022-01023-z

**Published:** 2022-02-22

**Authors:** Sean B. Wilson, Sara E. Howden, Jessica M. Vanslambrouck, Aude Dorison, Jose Alquicira-Hernandez, Joseph E. Powell, Melissa H. Little

**Affiliations:** 1 Murdoch Children’s Research Institute, Flemington Rd, Parkville, Victoria Australia; 2 Department of Paediatrics, The University of Melbourne, Victoria Parkville, Australia; 3 Garvan-Weizmann Centre for Cellular Genomics, The Kinghorn Cancer Centre, Darlinghurst, New South Wales Australia; 4 UNSW Cellular Genomics Futures Institute, University of New South Wales, Sydney, New South Wales Australia; 5 Department of Anatomy and Neuroscience, The University of Melbourne, Victoria Parkville, Australia; 6 Novo Nordisk Foundation Centre for Stem Cell Medicine, Copenhagen, Denmark

**Keywords:** Cell identity prediction, Human developing kidney, Kidney organoid

## Abstract

**Background:**

While single-cell transcriptional profiling has greatly increased our capacity to interrogate biology, accurate cell classification within and between datasets is a key challenge. This is particularly so in pluripotent stem cell-derived organoids which represent a model of a developmental system. Here, clustering algorithms and selected marker genes can fail to accurately classify cellular identity while variation in analyses makes it difficult to meaningfully compare datasets. Kidney organoids provide a valuable resource to understand kidney development and disease. However, direct comparison of relative cellular composition between protocols has proved challenging. Hence, an unbiased approach for classifying cell identity is required.

**Methods:**

The R package, *scPred*, was trained on multiple single cell RNA-seq datasets of human fetal kidney. A hierarchical model classified cellular subtypes into nephron, stroma and ureteric epithelial elements. This model, provided in the R package *DevKidCC* (github.com/KidneyRegeneration/DevKidCC), was then used to predict relative cell identity within published kidney organoid datasets generated using distinct cell lines and differentiation protocols, interrogating the impact of such variations. The package contains custom functions for the display of differential gene expression within cellular subtypes.

**Results:**

*DevKidCC* was used to directly compare between distinct kidney organoid protocols, identifying differences in relative proportions of cell types at all hierarchical levels of the model and highlighting variations in stromal and unassigned cell types, nephron progenitor prevalence and relative maturation of individual epithelial segments. Of note, *DevKidCC* was able to distinguish distal nephron from ureteric epithelium, cell types with overlapping profiles that have previously confounded analyses. When applied to a variation in protocol via the addition of retinoic acid, *DevKidCC* identified a consequential depletion of nephron progenitors.

**Conclusions:**

The application of *DevKidCC* to kidney organoids reproducibly classifies component cellular identity within distinct single-cell datasets. The application of the tool is summarised in an interactive Shiny application, as are examples of the utility of in-built functions for data presentation. This tool will enable the consistent and rapid comparison of kidney organoid protocols, driving improvements in patterning to kidney endpoints and validating new approaches.

**Supplementary Information:**

The online version contains supplementary material available at 10.1186/s13073-022-01023-z.

## Background

Single-cell RNA sequencing has transformed how we approach biological questions at the transcriptional level, facilitating accurate evaluation of cellular heterogeneity within complex samples, including entire tissues. When coupled with approaches for molecular lineage tagging [1] and computational approaches to analyse pseudotime [2–4] and RNA velocity [5, 6], gene expression in complex tissues such as the kidney can be studied at an unprecedented resolution. Despite these advantages, classification of cellular identity remains challenging and variable between datasets, even when analysing similar cellular systems. Currently, a common approach for identifying cell populations within single-cell data is to first cluster cells, compute differentially expressed genes between clusters and label clusters of cells based on expression of known marker genes [4, 7, 8]. The choice of clusters can be arbitrary, with users defining the number of clusters, thereby raising the potential for biases in the reproducibility of cell-type labels [9]. Placement of cells into a cluster relies on transcriptional similarity [10], hence there needs to be a large enough population with a distinct gene signature for this to occur. Cell clusters are also commonly defined based upon one or a few known differentially expressed genes rather than their global transcriptional signature. Finally, technical challenges such as batch variation can impact definitive cellular identification.

The application of single-cell profiling to developmental biology presents unique challenges due to the presence of intermediate cell types undergoing differentiation during morphogenesis. The mammalian kidney contains more than 25 cell types in the mature postnatal tissue, arising from a smaller number of progenitor cell types including nephron, stromal, endothelial and ureteric progenitors. Organogenesis is driven via reciprocal signalling and self-organisation with many intermediate transcriptional states that are less well defined, making the classification of cell types at the single-cell level both extremely useful but particularly difficult (reviewed in Little and Combes [11]). This is further complicated with hPSC-derived kidney organoid datasets. While protocols for differentiating kidney organoids from hPSC attempt to replicate in vivo kidney differentiation, they are limited and contain emerging non-specific, off-target, or synthetic cell types [12–15]. Here, unbiased classification of cellular identity is a computational challenge. Indeed, recent single-cell profiling of human fetal kidney (HFK) datasets have shown that the classical canonical markers for many cell identities within the kidney are not unique to these cell types but are also expressed at lower levels within other populations [15–18]. This makes cell classification in organoids more challenging when analysing gene expression of these markers in the single-cell clusters. The ability to robustly identify and classify cells in hPSC-derived organoid data is crucial to facilitate useful comparisons between datasets, particularly data generated using different differentiation protocols and cell lines as well as in response to mutation or perturbation. To compare between organoid protocols, studies have generated organoids for data integration and direct comparison [12, 14]. In other work, existing data has been integrated with new data with batch correction methods [19, 20] to identify conserved and unique features. These analyses help to improve and refine protocols towards a more accurate endpoint tissue.

One approach to cellular identification is to apply a small set of ‘known’ genes to identify clusters within a dataset based upon an existing reference dataset that has been accurately classified. Reviewing 13 published kidney or ureteric bud organoid single-cell RNA-seq datasets (Table 1), seven used a published HFK reference to find congruence with their clustered organoid cell populations either through integration or training a unique random forest classifier. However, many different HFK references were used across these publications while other analyses simply selected DE genes for classification without a reference source. Cell classification may be inconsistent when using various references containing different proportions of cells, possibly captured at different ages or regions of the tissue. Indeed, the most commonly used HFK reference only contained cells from the cortex of a 16-week kidney and hence was reported to contain few nephron cells and no ureteric epithelium [27]. There have been many tools developed to utilise reference data to classify a related query dataset, with scrna-tools.org [4] listing 110 tools in the ‘Classification’ category. These tools extract cell type information from an annotated reference and apply that to a query dataset. Most rely upon the user to supply the reference data and for those that supply a reference, none are directly relevant to hPSC-derived kidney organoids. The *R* packages *scTyper* [50] and *scClassify* [51] have models pre-trained on available kidney references; however, these are not ideal for the classification of the human developing kidney, due to training on mouse cell data (scClassify), or using gene sets of limited adult kidney cell types rather than developing kidney cell populations (scTyper). The browser-based tool, Azimuth, from HuBMAP (https://azimuth.hubmapconsortium.org/) [52] provides reference-based mapping for an uploaded single-cell gene expression matrix; however, the relevant available references are either human adult kidney or a human fetal development, the latter lacking the required granularity for the developing kidney. As such, there is a need for a tool that can be used to directly and accurately classify the cell types present within kidney organoids based on cell types within the developing human kidney.

**Table 1:** Summary of datasets used in this manuscript, including human fetal kidney and human kidney organoids

**Human fetal kidney**					
**Reference**	**Age (post coitum)**		**Sample details**		**Model usage**
Holloway et al. [21, 22]	16 weeks		Wedge biopsy including both medulla and cortex, 1 day 96 male and 1 day 108 female samples		**Train**
Hochane et al. [23, 24]	11, 13, 16 and 18 weeks		Week 9, 11, 13, 16 and 18 kidney pieces		**Train**
Tran et al. [25, 26]	17 weeks		Regions dissected from both inner and outer cortex		**Train**
	15 weeks		Regions dissected from both inner and outer cortex		**Test**
Lindstrom et al. [27, 28]	16 weeks		MARIS dissociation used to isolate cortical regions		**Test**
**Kidney organoids**					
**Reference & repository**	**Age (days)**	**Protocol**	**Sample information**	**ID**	**Initial classification**
Wu et al. [12, 29]	26	Takasato	4 batches of iPS and 2 batches of ES-derived organoids	Wu_T	Clustering & DE genes, Integration with self-generated adult snRNA dataset, Lindstrom [27] trained random forest classifier
	26	Morizane	3 batches of iPS and 1 batch of ES-derived organoids	Wu_M	
	34	Takasato	iPS-derived organoid extended culture	Wu_TO	
	7, 12, 19, 26	Takasato	Time course of iPS-derived organoids	Wu_TC	
	26	Takasato (modified)	2 batches of iPS-derived organoids with BDNF inhibition	Wu_TB	
Czerniecki et al. [30, 31]	21	Freedman	iPS- and ES-derived organoids, modified protocol for high throughput sequencing	Cz_F	Clustering & DE genes, Menon [32]
	21	Freedman (modified)	iPS- and ES-derived organoids, modified protocol for high throughput sequencing, VEGF addition	Cz_VEGF_F	
Howden et al. [13, 33]	18, 25	Takasato	iPS-derived organoids using E6 base media	How_T	Clustering & DE genes
Phipson et al. [20, 34], Combes et al. [15]	25	Takasato	iPS-derived organoids generated in two batches. Same dataset in both publications	Phip_T	Clustering & DE genes; Integration with Lindstrom [27]
Harder et al. [35, 36]	19,21	Freedman	ES-derived organoids, 6 datasets generated from the all organoids in a well, 3 separate batches	Har_F	Clustering & DE genes, integration and trajectory analysis with Menon [32]
	20	Freedman	A single ES-derived organoid isolated from a full well	Har_F_SO	
Subramanian et al. [14, 37]	7, 15, 29, 32	Takasato	iPS-derived organoids with 3 pooled replicates per time using iPS cell line designated “ThF”	Sub_T_L1	Clustering & DE genes, organoid trained random forest classifier, integration with Young [38], Lindstom [27] and self-generated kidney tissue
	7, 15, 29	Takasato	iPS-derived organoids with 3 pooled replicates per time using iPS cell line designated “AS”	Sub_T_L2	
Kumar et al. [19, 39]	25	Kumar (Takasato modified)	iPS-derived micro-organoid in suspension culture	Ku	Integration with organoid [15, 20] with clustering & DE genes
Low et al. [40, 41]	10, 12, 14	Low (novel)	Use three distinct phases of Wnt signalling, “defining”, “priming” and “patterning” the differentiating cells towards kidney organoid	Low	Clustering & DE genes
Tran et al. [25, 26]	16, 28	Morizane	ES-derived organoids	Tran_M	Clustering & DE genes individually & after integrating with self-generated kidney tissue
Lawlor, Vanslambrouck, Higgins et al. [42, 43]	25	Takasato	iPS-derived organoids generated by bioprinting. Organoids were compared with three different biophysical properties.	LVH_T	Clustering & DE genes compared to Hochane [24] trained machine learning model using scPred
Uchimura et al. [44, 45]	26	Takasato (modified)	iPS-derived organoids cultured following the Takasato protocol to day 7, before following Uchimura protocol to day 26	Uch_T	Clustering & DE genes, comparison to Wu [12] using pairwise Pearson correlation
	26	Uchimura (novel)	iPS-derived organoids generated by combining AIM and PIM differentiations in a 1:3 ratio at day 7 before culturing in modified maturation media novel to this protocol	Uch_U	
Wilson et al. (this publication)	25	Takasato (modified)	iPS-derived organoids generated in the same batch as Howden et al. [13] with RA treatment at day 12	Wil_TM	Direct comparison to existing organoids using DevKidCC
**Ureteric bud organoids**					
Howden, Wilson et al. [46, 47]	NA	Howden	Takasato iPS derived organoids dissociated and GATA3+EPCAM+ cells isolated. These cells cultured in ureteric epithelium promoting conditions.	HW_iUB	Seurat Label Transfer using reanalysed Holloway
Mae et al. [48, 49]	NA	Mae	Induced Ureteric Bud cultures	Mae_iUB	Clustering & DE genes

Here we have taken reference HFK datasets from three publications that span multiple ages and kidney regions (Table 1), performed individual annotations of the cells present based on prior information, then used all confidently classified cells to train classification models using the *R* package *scPred* [53], a generalisable method which has showed high accuracy in different experiments and datasets from multiple tissues, and considered a top performer in benchmarking studies [9]. We finally utilise established knowledge of kidney developmental biology to refine the classification of off-target cell types. The resulting model, referred to as *DevKidCC*, provides a robust and accurate classification of cells in novel single-cell datasets generated from developing human kidney or stem cell-derived kidney organoids. *DevKidCC* defines a model of cellular identity organised in a hierarchical manner to represent the key developmental trajectories of lineages within the developing kidney. The classification method is complemented with custom visualization tools in the *DevKidCC* package. This classifier was then used to investigate published kidney organoid datasets to compare organoid patterning and gene expression profiles across these datasets. We present a variety of applications of *DevKidCC* to the reanalysis of existing data. This analysis revealed differences in cell type proportions, nephron patterning and maturation between kidney organoid protocols. We also applied *DevKidCC* to investigate approaches for directed differentiation to one cell population, the ureteric epithelium, and dissect the effect of all-trans retinoic acid on nephron patterning and podocyte maturation. While *DevKidCC* is specifically trained on HFK for application to kidney organoid models, the development framework presented here could be applied for any tissue system to generate a cell classification model.

## Methods

### DevKidCC algorithm

*DevKidCC* (Developing Kidney Cell Classifier) is a function written in *R* designed to provide an accurate, robust and reproducible method to classify single cell RNA-sequencing datasets containing human developing kidney-like cells. The algorithm has two steps: data pre-processing and cell classification. Below we describe the development and utilisation of these steps.

### Data pre-processing

The required input is a scRNA-seq dataset as a *Seurat* [7, 8] object. The first step is extraction of the raw count matrix, which is then normalised by dividing the total expression of each gene by the total gene expression per cell then multiplied by a scale factor of 10,000 and natural log-transformed with pseudocount of 1.

### Cell classification

We generated a comprehensive developing kidney reference single-cell dataset by harmonising the raw data from multiple high-quality human fetal kidney datasets. The annotation of the reference included three tiers with increasing specificity, with a clear hierarchical structure between the tiers. This dataset was then used to train machine learning models using the *R* package *scPred* [53]. One model was trained for each node of identities within the classification hierarchy.

Utilising *scPred* [53] the classifiers were trained using the same parameters, with the relevant cells inputted for each. The feature space used was the top 100 principal components. The classifiers were trained using a support vector machine with a radial kernel using one round of harmonisation. The classifiers are stored as a *scPred* [53]object and can be used to classify cells within a *Seurat* [7, 8] object using the *scPred* [53] package. These classifiers will calculate the probability of a cell belonging to the trained identities within that classifier, giving a probability score between 0 and 1 for each identity. It will then assign an identity of the highest score above the set threshold or call the cell unassigned if no identity scores above the threshold. Classification is organised in a biologically relevant hierarchy so as to optimally and accurately identify the cellular identity of all analysed cells. All cells are first classified using the first-tier model, containing generalised lineage identities of stroma, nephron progenitors, nephron, ureteric epithelium and endothelium. After probability calculation using the first-tier model, cells that do not pass the threshold are classified as unassigned. The area under the AUROC and AUPRC to decide a threshold were determined using the *MLeval* R package from CRAN https://cran.r-project.org/web/packages/MLeval/index.html [54]. The threshold is set to 0.7 by default but can be adjusted by the user, which can be useful if the user wants to classify cells with decreasing degrees of probability. NPC cells were subjected to further investigation by subsetting and reclustering at a resolution level of 0.5 using *FindClusters* and identifying the percentage of cells expressing *PAX2* with clusters below 30% being relabelled as NPC-like. Cells assigned to stroma, nephron and ureteric epithelium are passed into a second tier of classification specific to these identities. It is important to note that at the second and third classification tiers, there is no thresholding, i.e., all cells are assigned an identity with no cells classed as unassigned. The second-tier ureteric epithelium model is trained on the tip, cortical, outer and inner medullary cell identities. The second-tier stroma model is trained on the stromal progenitors, cortex, medullary and mesangial cell identities. The second-tier nephron model is trained on the early nephron, distal nephron, proximal nephron, renal corpuscle and nephron cell cycle population. The distal nephron, proximal nephron and renal corpuscle are then further classified into more specific identities in a third tier of models. The third-tier distal nephron model is trained on early distal/medial cells, distal tubule and loop of Henle cells. The third-tier proximal nephron model is trained on early proximal tubule and proximal tubule cells. The third-tier renal corpuscle model is trained on parietal epithelial cells, early podocytes and podocytes. Each stage of the classification step is recorded as a metadata column, as is the final classification for each cell. All the probability scores and tier classifications are readily accessible within the *Seurat* [7, 8] object for further analysis.

### Comprehensive reference generation

Raw data was downloaded from GEO database from repositories GSE114530 [23] and GSE124472 [25] or provided to us directly by the authors, since made available at EMBL-EBI ArrayExpress under accession number E-MTAB-9083 [21]. The data as *CellRanger* output was read into *R* and processed using *Seurat* [7, 8] (v3.2.2), using *SCTransform* [55] for pre-processing. Clustering and manual annotation were performed on each dataset individually, referring back to the original papers and using established markers enriched in clusters to classify each cluster. Once annotated, datasets were integrated using *Harmony* [56] with 100 PCAs and 10000 variable features.

### Organoid gene expression database

All available single-cell RNA-sequencing kidney organoid datasets were downloaded (from Gene Expression Omnibus (https://www.ncbi.nlm.nih.gov/geo/) with accession numbers GSE118184, GSE109718, GSE119561, GSE114802, GSE115986, GSE132026, GSE124472, GSE152014, GSE161255, GSE152685, GSE131086) [25, 30, 33–35, 40, 42, 44, 46, 48]) and used to build a database. This database was generated by running *DevKidCC*, extracting summaries of the gene expression information at each classification tier and combining these into a formatted table. This database can be used to directly compare gene expression between existing datasets, also novel datasets classified using *DevKidCC*. The link to download this database is available at https://kidneyregeneration.github.io/DevKidCC/index.html [57].

### DevKidCC Kidney Organoid Gene Explorer shiny app

To make visualisation of the organoid database possible outside of using *R*, a shiny app was developed that provides an interface to interact with the organoid database using a modified *CompareDotPlot* function. This allows for an interactive way to visualise and analyse gene expression within published organoid datasets. This app is accessible at https://kidneyregeneration.github.io/DevKidCC/articles/ShinyApp.html [58].

### Downstream visualisation functions

To facilitate data visualisation and analysis of *DevKidCC* classified datasets, three customised functions were included in the package. *DotPlotCompare* is a modified version of the *DotPlot* function from the Seurat package. A gene expression profile of the reference is present within the function and can be used for direct comparisons to an existing or novel dataset. There is an option to visualise the organoid database within this function as well; the downloading instructions for this are available at the package Github repository (https://github.com/KidneyRegeneration/DevKidCC) [57]. The proportions of cells classified using *DevKidCC* can be visualised as a bar chart using the *ComparePlot* function. This can also take as input a gene and show the expression of that gene in each segment. The *IdentMeans* function produces summarises the contribution of samples to each population through a chart showing the mean and standard deviation/standard error of the mean.

### iPSC-derived organoid differentiation

The day prior to differentiation, CRL2429-MAFBmTagBFP2/GATA3mCherry human iPSCs [59] or CRL2429-SIX2EGFP [13, 59] were dissociated with TrypLE (Thermo Fisher Scientific, cat# 12563029), counted using a haemocytometer and seeded onto Laminin 521-coated (Biolamina, cat# LN-521-03) 6-well plates at a density of 50 x 10^3^ cells per well in Essential 8 (Thermo Fisher Scientific, cat# A1517001) medium. Intermediate mesoderm induction was performed by culturing iPSCs in TeSR-E6 medium (Stem Cell Technologies, cat# 05946) containing 4-8 μM CHIR99021 (R&D Systems, cat# 4423) for 4 days. On day 4, cells were switched to TeSR-E6 medium supplemented with 200ng/ml FGF9 (R&D Systems, cat# 273-F9-025) and 1 μg/ml Heparin (Sigma-Aldrich). On day 7, cells were dissociated with TrypLE, diluted five-fold with TeSR-E6 medium, transferred to a 15-ml conical tube and centrifuged for 5 min at 300 x *g* to pellet cells. The supernatant was discarded, and cells were resuspended in residual medium and transferred directly into a syringe for bioprinting. Syringes containing the cell paste were loaded onto a NovoGen MMX Bioprinter, primed to ensure cell material was flowing, with 100,000 cells deposited per organoid onto a 0.4-μm Transwell polyester membranes in 6-well plates (Corning). Following bioprinting, organoids were cultured for 1h in presence of 6μM CHIR99021 in TeSR-E6 medium in the basolateral compartment and subsequently cultured until day 12 in TeSR-E6 medium supplemented with 200 ng/ml FGF9 and 1 μg/ml Heparin. From day 12 to day 25, organoids were grown in TeSR-E6 medium either without additional supplement or with additional 5uM all-trans retinoic acid (Sigma-Aldrich, cat# R2625-100MG). Unless otherwise stated, kidney organoids were cultured until harvest at day 25.

### Flow cytometry

Prior to analysis, single-kidney organoids were dissociated with 0.2 ml of a 1:1 TrypLE/Accutase solution in 1.5-ml tubes at 37°C for 15–25 min, with occasional mixing (flicking) until large clumps were no longer clearly visible. 1 ml of HBBS supplemented with 2% FBS was added to the cells before passing through a 40-lM FACS tube cell strainer (Falcon). Flow cytometry was performed using a LSRFortessa Cell Analyzer (BD Biosciences). Data acquisition and analysis were performed using FACSDiva (BD) and FlowLogic software (Inivai). Gating was performed on live cells based on forward and side-scatter analysis.

### Whole mount immunostaining

Fixed kidney organoids were incubated in blocking buffer (PBS 1X donkey serum 10% triton X100 0.3%) at 4°C for 3h before adding primary antibodies against HNF4α (Life Technologies 1:300, cat# MA1-199), Nephrin (NPHS1 1:300, Bioscientific, cat# AF4269) and Claudin-1 (CLDN1 1:100, Thermo Fisher Scientific, cat# 71-7800) at 4°C for 2 days. After washing in PBS 1X triton X-100 0.1%, organoids were incubated in secondary antibodies 1:400 at 4°C for 2 days: Alexa fluor 405 donkey anti-mouse (Abcam, cat# ab175659), Alexa fluor 488 donkey anti-goat (Molecular Probes, cat# A11055) and Alexa fluor 568 donkey anti-rabbit (Life Technologies, cat# A10042). Samples were then washed before blocking at 4°C for 3h with PBS 1X mouse serum 10μg/ml triton X-100 0.3% and adding an APC-conjugated CD31 antibody (1:50, Biolegend, cat# 303115) at 4°C for 2 days. Finally, samples were washed and imaged in 50:50 glycerol:PBS 1X using a Dragonfly Spinning Disc Confocal Microscope (Andor Technology).

### Single-cell transcriptional profiling and data analysis

The novel dataset presented in this paper was generated from the same batch of samples presented in Howden et al. [13]. Human iPSC organoids were dissociated as described above (for flow cytometry) and passed through a 40-μM FACS tube cell strainer. Following centrifugation at 300 g for 3 min, the supernatant was discarded and cells resuspended in 50 μl TeSR-E6 medium. Viability and cell number were assessed, and samples were run across separate runs on a Chromium Chip Kit (10× Genomics). Libraries were prepared using Chromium Single-Cell Li sequenced on an Illumina HiSeq with 100-bp paired-end reads. Cell Ranger (v1.3.1) was used to process and aggregate raw data from each of the samples returning a count matrix. Quality control and analysis was performed in *R* using the *Seurat* package (v3.2.2). 1668 cells expressing more than 1500 genes and less than 30% mitochondrial genes passed quality control with means of 15100 for UMI count and 3697 for genes expressed. Classification was performed using *DevKidCC* (v0.1.6) as described in this manuscript.

## Results

### Generation of the model hierarchy for complete cell classification

**Fig. 1:**
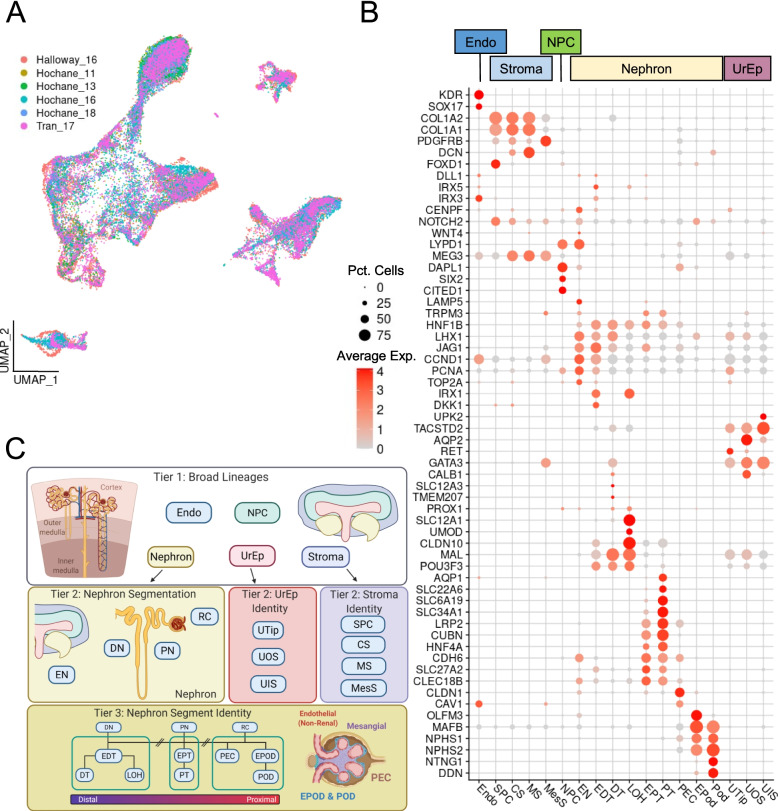
Generation of a comprehensive reference to train classification models. **A** UMAP visualisation of the integrated reference HFK datasets. **B** Expression of marker genes in the integrated reference shown by annotated identity. **C** Graphical representation of the *DevKidCC* model hierarchy and classification process. HFK human fetal kidney, Pct. percent of, Exp. expression

We first built a comprehensive reference dataset on which to train the probabilistic classification models. We used high quality HFK single-cell RNA-sequence datasets published in Hochane et al. [24], Tran et al. [26] and Holloway et al. [22] (Table 1). Samples ranged from 9 to 19 weeks’ gestation, across which time the developing human kidney undergoes both growth and maturation, with week 16 being most frequently represented. These references were originally annotated using clustering and cluster labelling using marker gene expression. One dataset was a recently published high quality HFK dataset [22] (8,987 cells) that included both medulla and cortex regions and including a 96-day male and 108-day female sample. Of note, this dataset contained ureteric epithelium, which had not been thoroughly analysed to this point [47]. This data was combined with data from 17,759 HFK cells ranging from week 11 to 18 of gestation [24] to increase the developmental range of the training set. A further 8317 cells from gestational week 17 which had been microdissected into the cortex, inner and outer medullary zones [26] were combined to complete the comprehensive reference single-cell RNA-sequencing HFK dataset. Cells from all datasets were integrated using *Harmony* [56] (Fig. 1A) before performing a supervised clustering and annotation, using the original annotations of each dataset as a guide. This led to a reference dataset containing three ureteric epithelial subpopulations including ureteric tip (UTip), outer stalk (UOS), inner stalk (UIS), four stromal subpopulations including stromal progenitor cells (SPC), cortical stroma (CS), medullary stroma (MS), mesangial cells (MesS), endothelium (Endo), the nephron progenitor cells (NPC) and the nephron including subpopulations of early nephron (EN), early distal tubule (EDT), distal tubule (DT), Loop of Henle (LOH), early proximal tubule (EPT), proximal tubule (PT), parietal epithelial cells (PEC), early podocytes (EPod) and podocytes (Pod) (Fig. 1B, C, Additional file 1: Fig. S1). Some of these populations were further classified in the original publications, including the DT being split into distal straight, distal convoluted and connecting segment or the classification of populations in relation to morphological features, such renal vesicle, comma shaped body and S-shaped body segmentation [24, 26, 47]. While morphologically there is a consistency in segment identification, this is less clear in single-cell data and has led to inconsistency in classification terminology. As such, here we have classified cell populations based on expression of known differentiation markers as cells take on a more distinct identity.

### scPred-derived models provide accurate classification of kidney cell types

The complex and dynamic nature of the developing kidney, with multiple cell lineages and waves of nephrogenesis, means that cells of many stages of differentiation can be present at all developing timepoints within the same single-cell data. This is one of the main challenges in classifying cells in the HFK single-cell data, as the cells are in transitional flux. The multiple lineages within the kidney also make classifying cell types difficult, as the differences between lineages mask the subtle differences in gene expression between cell types within a lineage, such as those of the epithelial sub-types. To minimise the impact of this transcriptional variance on classification, we took a hierarchal approach by using annotations at differing degrees of resolution to train three tiers of models (Fig. 1C). The models were trained with the package *scPred* [53] which utilises a machine learning approach to train sets of binary predictive models on a reference single-cell dataset. This model estimates the probability of a cell within a query dataset belonging to an identity group classified within the model. This has been shown to be a robust method to classify cells of a novel dataset based on a known reference [9, 43, 53]. The advantages of using *scPred* include ready integration with Seurat objects and the capacity to utilise many machine learning models available through the *caret* package. *scPred* provides ROC, sensitivity and specificity metrics using held-out training data for each binary classifier within a model which we used to benchmark multiple classes of models. A support vector machine with a radial basis kernel (svmRadial) and 100 principal components was used, with this performing equal to or better than a generalised linear model (glm) or neural network implementation (nnet) (Table S1A).

When implementing probabilistic models in practice, it can be beneficial to establish a threshold for a cell to be assigned an identity; however, this is difficult as organoids do not have a ‘ground truth’ for cell identity. While *scPred* provides ROC, sensitivity and specificity metrics using held-out training data for each binary classifier within a model (Table S1B), we wanted to investigate the model’s accuracy on organoid datasets. For this, we used two organoid single-cell datasets to test the binary classifiers within the tier 1 model which classified cells based on their lineage; nephron progenitor cells (NPC), nephron, ureteric epithelium (UrEp), stroma and endothelial. The Howden et al. [13] organoids were used as we had access to the original annotation showing a representation of all key cell types, while the Uchimura et al. [45] dataset was reported to be enriched for the UrEp population. We used the standard clustering pipeline to reproduce Uchimura annotation from the original publication. While the AUROC was 0.93 or higher for all tests, the AUPRG curves showed a much faster drop off in precision for the Howden et al. [13] test than Uchimura et al. [45] (Additional file 1: Figure S2A). Using the performance of the Nephron, NPC, UrEp and Stroma binary classifiers in these tests led to setting a default threshold of 0.7 for the tier 1 model’s classification with all cells having a maximum probability below this remaining ‘unassigned.’

**Fig. 2:**
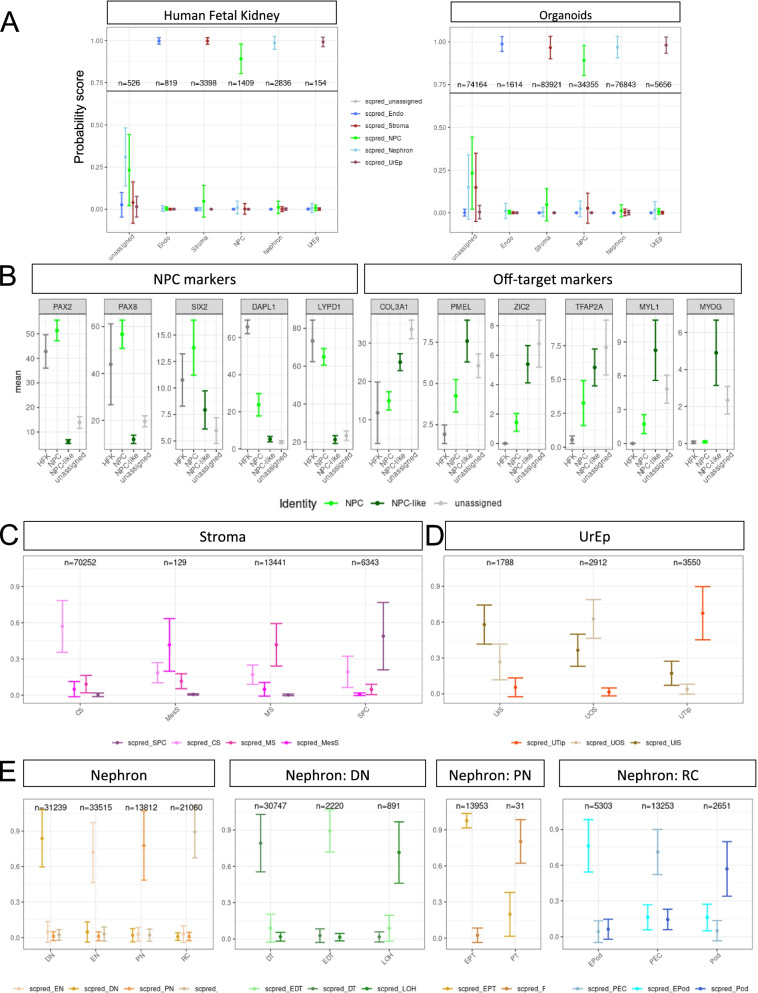
DevKidCC accurately classifies human fetal kidney data. **A** Probability score distributions for the tier 1 classifier for both human fetal kidney (left) and organoid (right) data, grouped by tier 1 classification. **B** Mean number of cells expressing shown genes, grouped by HFK NPCs, organoid NPCs, organoid NPC-like and organoid unassigned populations. **C** Probability score distribution for the tier 2 stroma classifier on all organoids. **D** Probability score distribution for the tier 2 UrEp classifier on all organoids. **E** Probability score distribution for the tier 2 and 3 nephron lineage classifiers on all organoids

We next investigated the probability scores of the model on all freely available published organoid datasets (371,570 cells from 58 samples) ranging from 7 to 32 days of culture and two human fetal kidney datasets including the frequently used Lindstrom et al. [27]. While the distribution of the maximum scores for cells in the HFK and organoid datasets showed very similar patterns, organoids showed a lower mean and larger SEM (Fig. 2A). A two-sample *t* test comparing the HFK and organoid probability scores of ‘end-stage’ organoids, i.e., those beyond 18 days of culture, indicated significant differences between the assigned nephron (*p* < 2.3 x 10^-36^), UrEp (*p* < 2.9 x 10^-25^), stroma (*p* < 2.3 x 10^-308^) and NPC (*p* < 2.3 x 10^-308^). The model classified between 60.8% and 92.0% of ‘end-stage’ organoids, while the HFK samples had >90% classification (Fig. 2A). The ‘unassigned’ cells may represent non-renal off target cell types not normally present in HFK or cells in which identity is not sufficiently strong for definitive classification. When applied to the dataset of Lindstrom et al. [27], this model classified 90.4% of the 2945 cells that passed quality control, while the remaining cells expressed markers for immune cells (*HLA-DRA*, *CCL3*, *SRGN*) which are not represented in the model and so were not assigned an identity (Additional file 1: Figure S2B,C). 14 cells (0.5%) were classified as UrEp, positioned at the tips of one end of the nephron cluster. The nephron cells nearest to the UrEp population were further classified as DN epithelium (not shown). While these two cell populations arise from distinct precursors, they share a similar transcriptional profile, making them difficult to distinguish at single-cell level [15–18, 47]. The ability to identify and classify these two populations separately, even with a small contribution of one population within a dataset, demonstrates the power of using *scPred-*derived models. The expression of marker genes used by Lindstrom et al. [27] to annotate cell identities were shown as enriched in the same populations classified using *DevKidCC* (Additional file 1: Figure S2C), affirming the accuracy and relevance this classification method.

Some samples showed classified NPC populations with enrichment for ‘off-target’ markers of muscle (*MYL1*, *MYOG*) neural (*ZIC2*) and melanin-expressing (*PMEL*) populations, in line with previous reports (Additional file 1: Fig. 2D). This is particularly relevant as the Howden et al. [13] dataset used for model evaluation is one of these. One theory for the generation of these cell types is misdirected differentiation potentially from a shared progenitor with the NPC cells that arise, which may explain the similarity to the NPC profile and high probability score. *PAX2* has been shown as a gene that marks the ‘lineage boundary’ between the NPC and stromal populations in vivo [60], as well as being an early marker of the nephron lineage [61]. There is evidence, however, that *PAX2* is dispensable for in vitro nephron formation [62], once again highlighting differences between in vivo and in vitro systems. We investigated the expression of *PAX2* in the NPC classified cells and found a correlation between *PAX2* expression and other key markers of NPCs such as *LYPD1* and *SIX2*. There is also an inverse correlation between *PAX2* expression and off-target markers of muscle, neural and melanin-expressing cells (Fig. 2B). Incorporating this biological knowledge to refine the classification of NPCs, we included a step to subcluster and screen for *PAX2* expression, with subclusters having less than 30% of cells express *PAX2* being relabeled as ‘NPC-like’ (Fig. 2B). These cells may have the potential to undergo nephrogenesis if correctly induced however lack a clear in vivo NPC transcriptional signature, making them likely in vitro artefacts arising in this system.

The cells classified as nephron, stromal and UrEp underwent the further stage/s of classification. The stromal and UrEp population utilise one further classifier, classifying them into stromal subsets of SPC, CS, MS and MesC (Fig. 2C), while the UrEp population is further classified into UTip, UOS and UIS identities (Fig. 2D). The nephron population however has additional segmentation and requires two further stages of classification (Fig. 2E). A summary of the *scPred* provided AUROC, sensitivity and specificity metrics generated using held-out training data for each binary classifier within a model, which range between 0.879 and 1.000, are provided in Additional file 3: Table S2. However, lack of a ‘ground truth’ for organoid identity makes it difficult to precisely evaluate model performance using held out in vivo data. To complement that analysis, we compare the probability scores for all tests within an identity (Fig. 2C–E). These results highlight the accuracy of these additional models, particularly within the nephron cell identities.

These models are utilised in a hierarchical method of classification provided in a single-call wrapper function *DKCC()* within the R package *DevKidCC*, taking an input a *Seurat* object. To determine cells in the first tier, a probability threshold of 0.7 is set while at all other tiers the threshold is removed. This enables all cells that are classified at the top tier to be given an assigned identity regardless of the highest degree probability predicted by the lower tier models. Further investigation of the calculated probability can be interrogated as every cell has a record in the metadata of the scores from each classification. No preprocessing is required as *DKCC()* utilises Seurat’s *NormalizeData()* function for data normalisation. The recommended pipeline is to read in raw counts data using the *Seurat* pipeline, filter out poor quality cells and then run *DKCC()*. The classifications for each tier and the final identities can be accessed within the metadata slot for further investigation. The package contains custom in-build functions *ComparePlot*, *DotPlotCompare* and *IdentMeans* to investigate the cell populations within the classified sample.

### DevKidCC classification rapidly and accurately reproduces published annotations

**Fig. 3:**
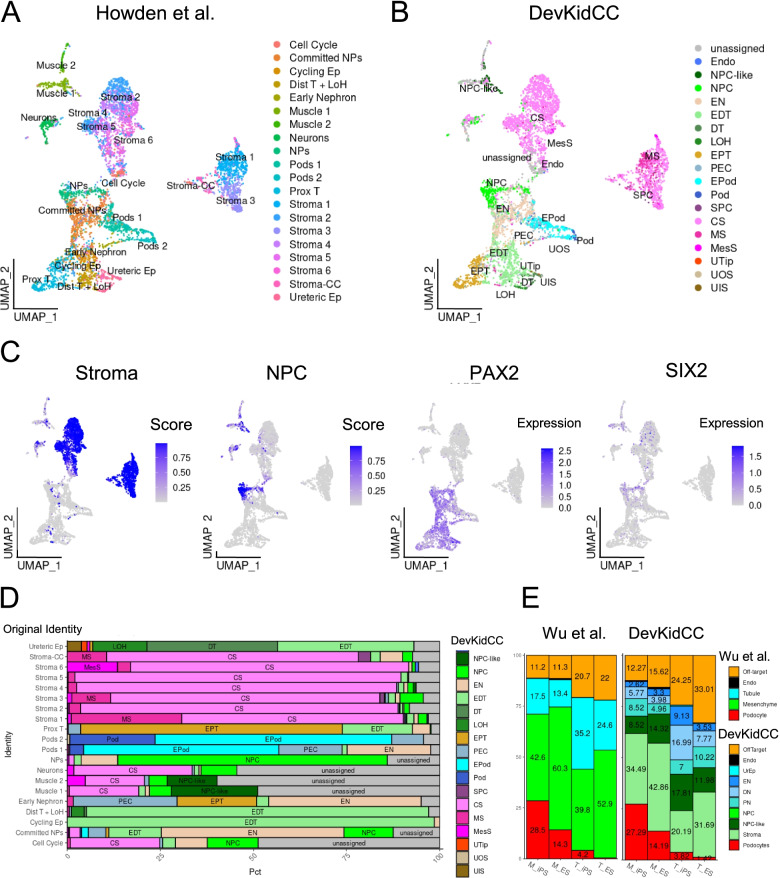
DevKidCC classification of organoid datasets. **A** UMAP representation of the original classification of the Howden dataset. **B** DevKidCC classifications of the same dataset. **C** UMAP representation of Howden dataset showing Stroma and NPC prediction scores, *PAX2* and *SIX2* expression values. **D** ComponentPlot showing the reclassification of the Howden dataset including distinguishing between NPC and NPC-like populations. **E** The original (left) and comparative DevKidCC (right) classification of data published in Wu. NPC nephron progenitor cell

To investigate the utility of using this package on real-world data, the *DevKidCC* classification of two published kidney organoid single-cell datasets was compared to their original cluster-based annotations. Howden et al. [13] contained samples from two differentiation timepoints; intermediate (18 day) and late (25 day) stage organoids while Wu et al. [12] contained day 26 organoid datasets from two distinct protocols for deriving kidney organoids, labelled as Takasato [63] and Morizane [64] after the original authors. Within the Howden et al. [13] and Wu et al. [12] data, 76.0% and 61.1% of cells were assigned using *DevKidCC*, respectively. Within the Howden et al. [13] dataset, all original clusters contained cells that were reclassified as unassigned, with the largest contribution being from clusters previously annotated as the neuron and muscle, illustrating the specificity with which the model classifies renal cell types (Fig. 3A).

Both stroma and NPC are mesenchymal cell types. The mesenchymal cells present within kidney organoids have been difficult to accurately classify due to their gene expression profiles being different to those of characterised developing kidney stroma [15]. There was some overlap in the distribution of cells with high probability for both stroma and NPC (Fig. 3C). Cells within organoids that share expression profiles both with stroma and NPC have been previously noted [16] and may arise as an in vitro artefact. The *PAX2* lineage differentiation between NPC and NPC-like cells is clearly shown when comparing the high probability NPC cells expressing *PAX2* being localised to the larger ‘nephron’ cluster while the high probability NPCs not expressing *PAX2* are separately localised in distinct clusters (Fig. 3C). The expression of SIX2, the gold standard in vivo NPC marker, is shown for comparison and has a wider distribution including other mesenchymal cell types labelled as Stroma, in line with the initial publication of this data (Fig. 3C). *DevKidCC* reclassified ~25% of cells originally annotated as ‘muscle’ and ‘neural’ as NPC or NPC-like, while also reclassifying some as stromal cells. The off-target populations were noted to share expression of key NPC markers such as *SIX2* and *SALL1* [13] indicating there is some transcriptional similarity. Within the nephron, cells previously identified as ‘Committed and Early Nephron’ due to the expression of both committed NPC (*LYPD1*) and early nephron (*LHX1, JAG1*) markers within this cluster were reclassified by *DevKidCC* to distinguish between the two cell populations (Fig. 3D). The previous analysis of the Howden et al. [13] data identified seven clusters as stromal (Fig. 3A, D), of which almost all of those assigned an identity using *DevKidCC* remained classified as a stromal sub-type (Fig. 3D).

To further examine the capability of *DevKidCC* classification, we analysed organoid datasets from Wu et al. [12] generated from either embryonic (ES) or induced pluripotent (iPS) stem cells using two different protocols [12]. Using *DevKidCC* with default parameters, we were able to rapidly reproduce the initial classification of these organoids, accounting for the differences in the nomenclature (Fig. 3E). This classification identified an increased population of cells not matching the reference (termed ‘unassigned’) compared to the original annotation. Here, *DevKidCC* could again distinguish kidney cells from likely off target cell types, such as the originally reported neural population, that may represent artefacts of in vitro culture [12, 13]. This demonstrates how *DevKidCC* provides a consistent and measurable benchmark for kidney cell classification in organoids that can be applied to all data, enabling direct and relevant comparisons. Together these reanalyses demonstrate the accuracy with which *DevKidCC* can classify renal cell types within organoid datasets.

### DevKidCC provides a method for direct comparison between protocols

**Fig. 4:**
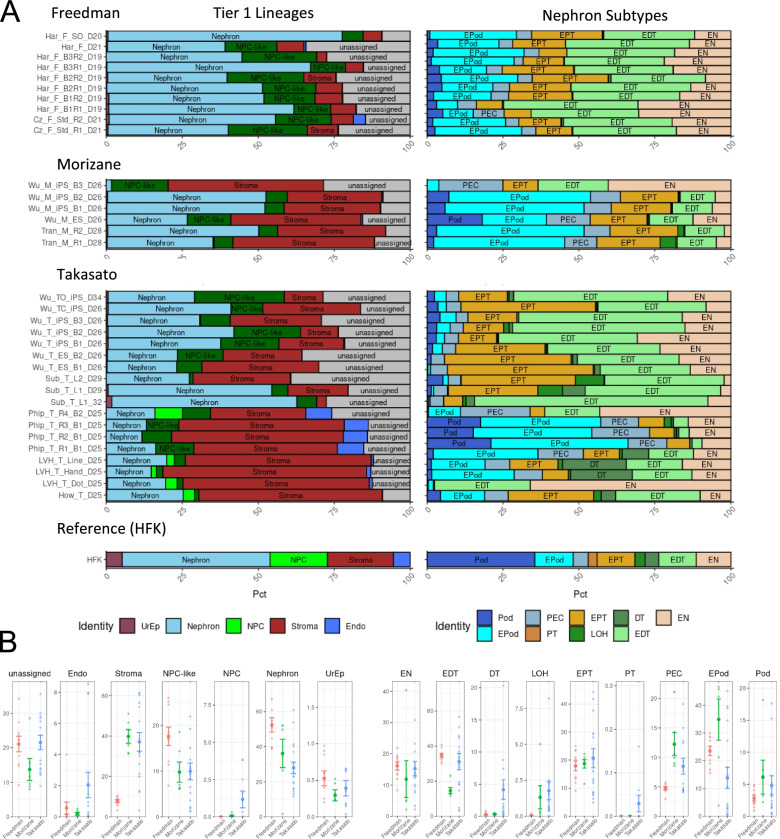
Direct comparison of organoids generated from different protocols. **A** Proportion of cell type contribution for all end-stage samples of Freedman, Morizane and Takasato protocols at the first tier of classification (left) and the breakdown of nephron sub-types (right), with the reference for comparison. **B** Direct comparisons of percentage cell contributions between these protocols. Pct percent of total cells

A major challenge for the field has been to compare between datasets generated from different labs, lines, batches or from different protocols due to differences in the analyses that were used. This is particularly pertinent given the use of several distinct protocols for generating kidney tissue from hPSCs (see Table 1). Direct comparisons between studies and protocols requires an integration of all existing samples to allow re-clustering and differential gene expression analysis on the combined dataset. This is challenging due to the noise between samples, the majority of which relates to technical or batch effects [20] that can confound biological variations of interest during data integration [65]. To avoid these challenges, *DevKidCC* was used to directly identify all cell types present within multiple datasets enabling direct comparisons without the need for integration. As *DevKidCC* will compare all cells to the same comprehensive reference, the biological information for each sample can be directly compared without prior dimensional reduction and clustering. To demonstrate this, we applied *DevKidCC* to all available single cell kidney organoid datasets (summarised in Table 1) irrespective of the cell line, organoid age, differentiation protocol or laboratory. This comprehensive analysis allows a direct comparison of cell proportions across all samples at each tier of classification. We first focused on end stage organoids from the three main differentiation protocols represented in the literature, Takasato et al. [63], Morizane et al. [64] and Freedman et al. [66] (Fig. 4A). This immediately showed variation in the proportions of ‘unassigned’ cells across all datasets and the lack of nephron maturation even in the oldest organoids regardless of protocol. The maturation of nephron cell types was limited in all protocols and samples, although the Morizane [64] protocol produced organoids with the highest number of cells reflective of a more mature podocyte stage. While there were a small number of mature podocytes, there were almost no mature proximal tubule cells generated with any organoid protocol, with cells rather being classified as less mature EPT with expression of proximal markers such as *CUBN*, *LRP2* and *HNF4A* but lack the specific solute channels such as *SLC47A1*, *SLC22A2* and *SLC22A8* (Additional file 1: Figure S3). In clustering-based analyses, these cell populations are often split into two or more groups which are interpreted to have varying degrees of maturation, whereas the *DevKidCC* classification indicates that these are mostly immature. There is noticeable variance between publications generating organoids from the same protocol, concurring with earlier studies showing that batch differences are a notable source of variation [12, 20]. We performed two analyses of the unassigned populations, grouped by protocol. The most conserved differentially expressed genes between samples of each protocol were inputted into the ToppFun browser, with the Takasato protocol [63] and Morizane protocol [64] derived populations showing similar upregulated pathways such as skeletal system development and extracellular matrix organization, while the Freedman protocol [66]-derived populations showed an enrichment for neural system pathways (Additional file 1: Figure S4A). A second independent analysis used the Azimuth web browser to annotate these cells using the human fetal development reference. This analysis predicted the identity of each cell, with the strongest probabilities falling in the skeletal muscle and satellite cell categories in all protocols, with a range of other cell types being predicted, including populations of metanephric and ureteric cells of the kidney, various stromal populations and some neural subtypes (Additional file 1: Figure S4B). Interestingly, 37.47% and 23.38% of these cells derived from Morizane [64] and Freedman [66] protocols, respectively, were assigned metanephric while only 0.89% of Takasato [63]-derived cells. This analysis highlights the variation in the transcriptomic profiles present within organoid populations and the challenges in identifying those cell types even with the best available analysis pipelines.

*DevKidCC* analysis revealed differences in cell proportion and nephron patterning between organoids generated with different protocols. The proportion of cell populations within the reference HFK is unlikely to be representative of the ratios within a developing kidney due to the different methods used to collect samples; however, as organoid samples are typically a whole sample dissociation, we can infer some information comparing between these. Organoids generated using the Freedman protocol [66] show a small stromal population in comparison to other protocols while containing more early-stage nephron cells, although this may be indicative of the slightly younger age of these organoids. In the Morizane protocol [64] organoids, we identify limited distal tubule cells, with less than 25% of the nephrons classified as distal whereas the Takasato [63] and Freedman [66] protocols show more evenly segmented nephron components. The Takasato protocol [63] generates the most distal tubule (Fig. 4B), including some cells classed as a more mature DT segment as well as an *SLC12A1* expressing Loop of Henle population. The DT expressed *GATA3* and *TMEM52B* but lacked the distal convoluted tubule specific marker *SLC12A3*, although in some cases the connecting segment specific marker *CALB1* is expressed (Additional file 1: Figure S4). This would indicate that the connecting segment, which represents the most distal region of the nephron and which invades and fuses into the ureteric tip to form a contiguous tube, is being generated in some organoids. This is promising as it would indicate that there is the potential to promote fusion of these nephrons to any separately induced collecting duct structure, potentially enabling kidney tissue engineering. In summary, while nephrons are forming and showing evidence of patterning and identifiable segmentation in all protocols, their relative proximo-distal patterning and evident immaturity will impact their utility for disease modelling and drug screening studies.

### Identifying nephron progenitor cell variation between protocols

To further investigate relative gene expression between datasets, we extracted gene expression profiles and proportions of cells in each classified population, in all available organoid datasets (see Table 1) and the comprehensive reference. A modified version of the *DotPlot* function from the *Seurat* [7, 8] package was included to directly compare gene expression between datasets and the reference. The NPC are a crucial population when considering kidney organoids as in vivo they give rise to the entirety of the nephrons [67], the functional unit of the adult kidney. Our classification system has highlighted the difference between NPC and NPC-like cells that arise during in vitro differentiation; however, we sought to further investigate the NPC population within organoids. The direct comparison between kidney organoids (Fig. 4A, B) revealed substantial variation in the proportion of NPCs, which we further investigated by applying the function *DotPlotCompare* (modified *DotPlot* from the Seurat package) to visualization relative gene expression in NPCs across all protocols.

The nephron develops from NPCs which are a heterogeneous population of mesenchyme that undergo a mesenchyme to epithelial transition (MET) in response to signals from the ureteric epithelium, giving rise to the entire nephron epithelium [67, 68]. In vivo analysis has shown markers like *SIX1*, *SIX2*, *CITED1*, *DAPL1* and *LYPD1* are expressed in this population and can be used to reliably identify these cells from the surrounding stromal mesenchyme in situ [17, 27]. These markers have also been used to identify the NPC populations of cells in both HFK and organoids in single-cell datasets. When analysing NPC from within the reference HFK dataset using *DevKidCC*, we can see that 44.9% of cells express *SIX2*, 56.3% express *SIX1*, 53.3% *CITED1* while over 70% express *DAPL1* and *LYPD1*. Importantly, the kidney is the third excretory organ to arise during development. The final kidney is comprised of metanephric nephrons and is preceded by the pronephric and mesonephric tubules [69]. These arise in an anterior to posterior manner, which is reflected in their respective HOX codes. Within the HFK reference data set, the NPCs that give rise to the metanephric nephrons express a posterior HOX code, particularly the HOX10 and HOX11 paralogues [70, 71]. The posterior HOX genes are expressed, with *HOXA10* most abundant and *HOXC10*, *HOXD10*, *HOXA11* and *HOXD11* at lower levels and in less cells. The heterogeneity of gene expression within this population could result from data sparseness, dropout levels and capture bias. It may also be explained by transcriptional bursting [72], where genes are not constantly being transcribed and so the sample harvesting may occur during a transcriptional lull. However, this does provide a true reference for comparison to the expression profiles expected within these cell populations in organoids.

**Fig. 5:**
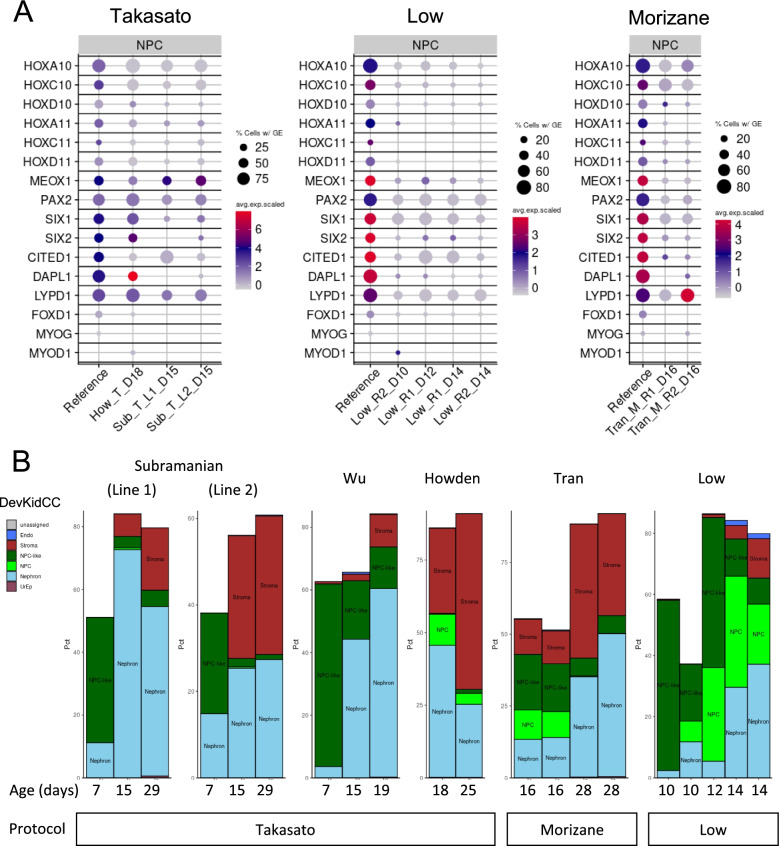
Direct comparison of NPCs generated from different protocols. **A** Gene expression of all samples day 16 or less, grouped by protocol. **B** Proportions of classified cells for groups of samples with time-course information (*y*-axes unique to each plot). NPC nephron progenitor cell

When we compare organoid NPCs to the HFK reference, we again note variance between publications and protocols. While the majority of organoid datasets are end-stage and thus are largely depleted of NPCs, this analysis confirmed previous studies showing a population of NPCs, sometimes referred to as mesenchymal progenitors, can remain. Organoids containing more than 30 NPC cells were analysed for the expression of NPC markers. Takasato protocol [63]-derived NPCs show expression of the posterior HOX code and in many samples known NPC markers, while the Morizane protocol [64], Freedman protocol [66] and Low protocol [41]-derived NPCs lack expression of posterior HOX genes, but do express some expected NPC markers, most abundantly *LYPD1* and *SIX1* while these are limited in Morizane protocol [64] and Freedman protocol [66]-derived NPCs, with almost no *SIX2*, *CITED1* and *DAPL1* present*.* Analysis of NPCs in end-stage organoids is not optimal as the prolonged culture may cause some transcriptional variability. Indeed, differences in mouse NPCs across developmental time have been characterised [73]. However, these traits are observed in samples of younger organoids and even monolayer time-points (Fig. 5A). In the ‘unassigned’ and ‘NPC-like’ populations generated in organoids, expression of the muscle markers, including *MYOG* and *MYOD1* was sometimes evident. A subset of individual cells within such a published ‘muscle’ cluster [13] were re-classified by *DevKidCC* as NPC but do show expression of these muscle genes (Fig. 5A, Additional file 1: Figure S3). Indeed, muscle gene expression is detectable in kidney organoid clusters previously labelled as NPC from multiple protocols and publications [12–15, 31]. However, there is no evidence for the expression of these genes in the HFK reference, suggesting that their consistent expression in organoid populations is an artefact of the in vitro culture conditions. During in vivo kidney development NPCs undergo a balance of self-renewal and commitment to nephrogenesis, allowing for ongoing waves of nephron formation leading to an average of 1 million nephrons per human kidney [74]. However, developing organoids in vitro undergo limited nephrogenesis, leading to 10 to 100s of nephrons per organoid [13]. This variation is presented grouped by experiment and timepoint, with the NPC percentage decreasing with time (Fig. 5B). The Low et al. [41] samples show an NPC peak at day 12 before a decrease by day 14 coinciding with the appearance of nephron populations. As organoids age, the NPC and NPC-like populations decrease or deplete while nephron and stromal populations increase, likely at a faster rate than is required to maintain an NPC population for ongoing nephrogenesis. In summary, we have identified an in vitro culture artefact muscle gene signature within the NPC population present across multiple protocols, giving a target to modulate for improving NPC identity within organoids. We also identify a decrease in expression of key NPC genes, including *SIX2* which in mouse is believed to govern self-renewal, indicating a potential cause for the lack of ongoing nephrogenesis in vitro*.* This analysis demonstrates how using *DevKidCC* to classify and directly compare all published organoids datasets can improve our understanding of NPC population generated across multiple kidney organoid protocols.

### Application of DevKidCC to investigate the impact of retinoic acid on kidney organoid maturation

**Fig. 6:**
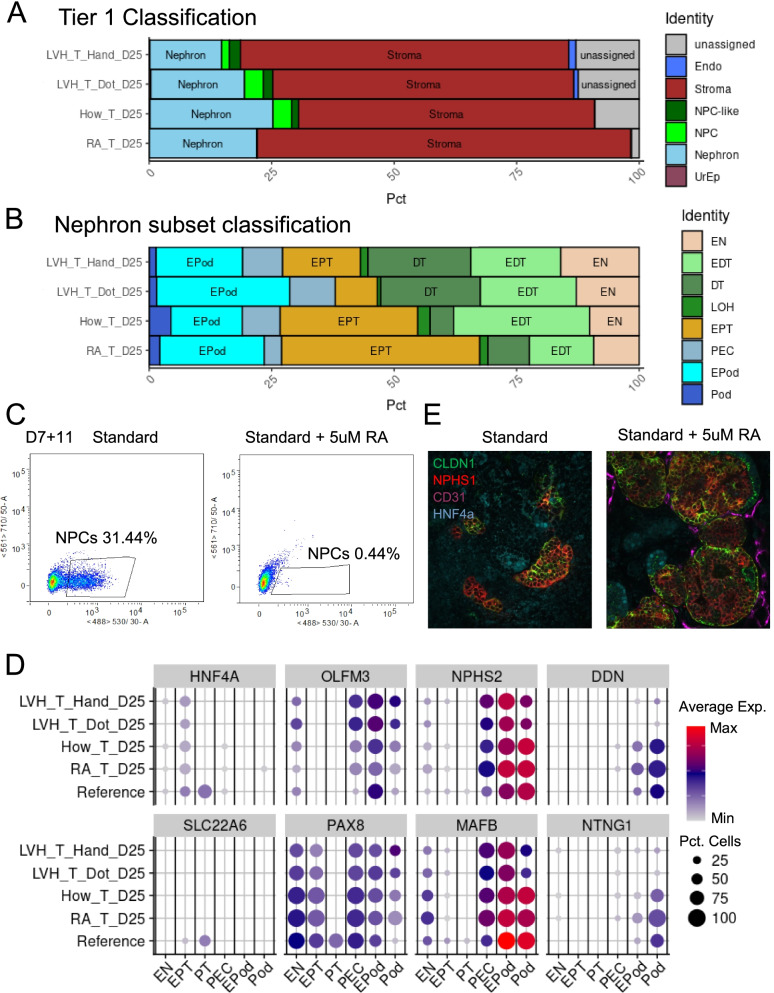
Effect of retinoic acid when added to mid-stage organoids. **A** ComparePlot comparison of control and treated organoids datasets grouped by tier 1 lineage classification. **B** ComparePlot comparison of control and treated organoids showing the nephron identity grouped by cell sub-type. **C** FACS plot showing effect of RA addition on SIX2+ cell population. **D** Expression of PT and Pod gene markers in control and treated organoid datasets. **E** Expression of CLDN1 in control (left) and treated (right) organoids. RA retinoic acid, PT proximal tubule, Pod podocyte

Accurately identifying the cell types present within an organoid is crucial for the analysis of disease states or the optimization of the differentiation protocols. To evaluate the application of *DevKidCC* in analysing functional differences between methods, we analysed unpublished data in which kidney organoids from the same starting cell line generated from the same batch were treated with 5μM retinoic acid (RA) after removal of all other growth factors at day 12 of the Takasato protocol [63] to promote maturation. Mammalian nephrogenesis in vivo occurs in waves with new nephrons constantly forming up to 36 weeks gestation [75, 76] in humans and into the first week of life in mice [77]. This is facilitated by the presence of a peripheral nephrogenic niche within which the NPC balance self-renewal versus nephron commitment. Once differentiated, NPCs exist throughout the duration of organoid culture and deplete with time, although a population does remain in mature organoids able to undergo nephrogenesis when induced with a canonical Wnt agonist [13] (Fig. 4A). Retinoic acid signaling plays many roles in kidney development depending on spatiotemporal expression [78–80] and is also known to promote the differentiation of progenitor cell populations [81]. We investigated adding all-trans retinoic acid to organoids at multiple time points to see what effect this would have on organoids. The addition of 1–5 μM RA before day five of 3D organoid culture, substantially impaired nephron formation, whereas addition at day five onwards led to organoids with fully segmented nephrons similar to organoids without RA (data not shown). The *DevKidCC* classification identified an increase in the percentage of classified stromal cells, seemingly at the expense of the ‘unassigned’ population. In contrast to control organoids from the same batch (How_T_D25) [13] and organoid datasets of the same line, age and differentiation protocol (LVH_T_Hand_D25 and LVH_T_Dot_D25) at day 25, the addition of RA resulted in a complete depletion of NPC cells (Fig. 6A). While the percentage of nephron cells did not change, there was a shift towards early proximal tubule (EPT) (Fig. 6B). The addition of RA also drove an increase in the presence of classified stroma at the expense of unassigned cells. These comparisons indicate that RA caused the depletion of NPCs, expansion of renal stroma and proximalisation of nephrons within forming organoids. To confirm if NPCs were indeed depleted by RA addition, organoids were generated using a SIX2^EGFP^ reporter line [13, 59] with and without the addition of RA and analysed using flow cytometry after 7 days. The control organoids had 31.44% EGFP+ cells while the organoids with RA had less than 0.5% (Fig. 6C). This confirms that RA acts directly or indirectly on the NPC population, forcing them to either undergo commitment to form nephrons or differentiate away from NPC identity down a stromal pathway.

To investigate the maturation of the nephrons we visualized maturation markers both proximal tubule and podocytes using the *DotPlotCompare* function within the package. While there was an increase in the percentage of nephron cells identified as proximal tubule, this was entirely EPT and there was no evidence of increased maturation at a transcriptional level with no expression of mature PT genes like *SLC22A6*. (Fig. 6D). There was an increase in the expression of podocyte maturation genes such as *DDN*, *NTNG1* and *NPHS2* with RA addition, corresponding with a decrease in *OLFM3* and *PAX8* expression as predicted for genes expressed in immature podocytes but downregulated with maturation [24]. Immunofluorescence showed PEC marker CLDN1 had improved localization to the epithelial cells surrounding the podocytes, which is the normal location of PECs (Fig. 6E). The expression of both PEC and podocyte markers in cells assigned to all three renal corpuscle identities is consistent with the previous analysis of these populations and may indicate that the delineation of specific gene signatures within these cells is not yet occurring.

### Analysis of existing protocols for the development of ureteric epithelium

The ureteric epithelium in the mammalian kidney arises as a side branch of the mesonephric duct that grows into the presumptive kidney mesenchyme [82]. Hence it has been suggested that it is not possible to generate ureteric epithelium using the same differentiation protocol able to generate the nephron lineages [83]. Single-cell analyses have recently revealed the significant transcriptional congruence between the distal nephron and the ureteric epithelium in both human and mouse [12, 16]. It has also been established that distal nephron from standard organoids remains plastic and can be induced to adopt a ureteric epithelial fate [18]. To date, a number of protocols have been published that report the generation of ureteric epithelium [45, 47, 49, 83, 84] both from single monolayer differentiations generating both nephron and ureteric segments, or the isolation of cellular fractions that are then cultured separately to form ureteric epithelium. While organoids generated from a single differentiated monolayer have been reported to contain both nephron and ureteric lineages [13, 45, 63], this was due predominantly to expression of markers like *GATA3* and *HOXB7* which have been further identified as expressed in distal nephron segments [16, 18, 47].

**Fig. 7:**
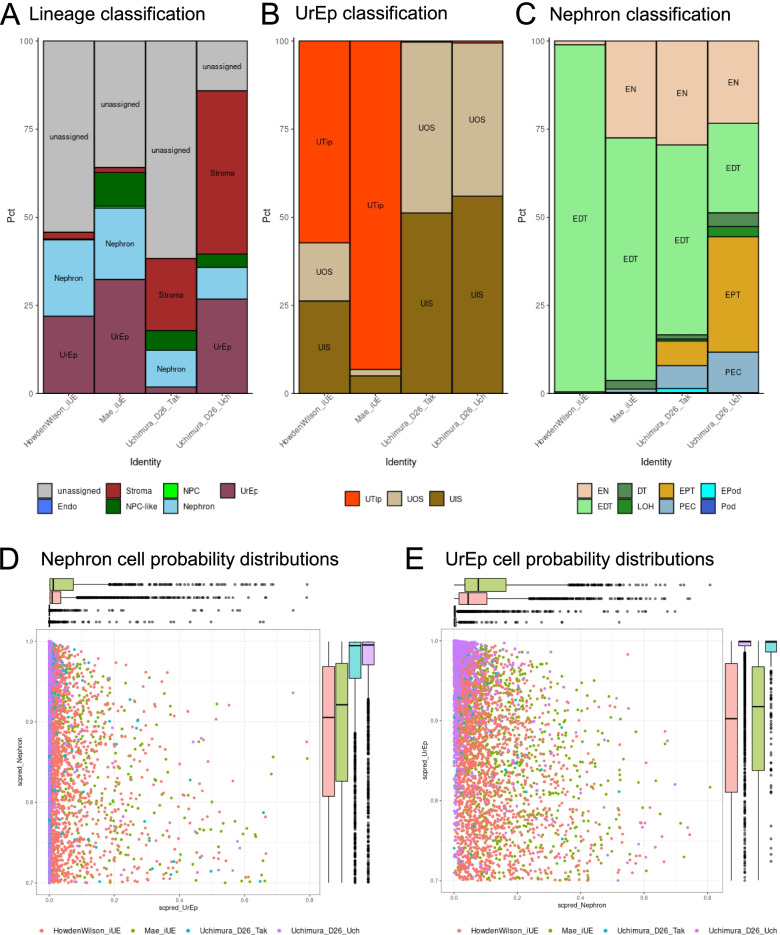
Classification of ureteric cell types in organoid and targeted cultures. **A** The DevKidCC classification for the in vitro samples targeting UrEp culture. **B** Further classification of all UrEp cells from previous panel. **C** Further classification of all nephron cells from panel **A**. **D** Comparison of nephron and UrEp probability scores for all nephron classified cells. **E** Comparison of nephron and UrEp probability scores for all UrEp classified cells. UrEp ureteric epithelium

As *DevKidCC* had shown an accurate delineation of UrEp and Distal Nephron in the HFK samples (Additional file 1: Figure S2B), we investigated the *DevKidCC* classification of four single cell samples claiming substantial UrEp generation using different approaches; one from a targeted UrEp differentiation [49], one from UrEp that had been derived from DN [47] and two from organoid samples generated either using the Takasato protocol or a mixed-culture approach with further UrEp-enhancing culture conditions [45] (Table 1). *DevKidCC* classified 32.41%, 21.96%, 1.87% and 26.81% of all sample cells as UrEp, respectively (Fig. 7A). The targeted UrEp cultures retained a more proliferative tip-like identity while the organoid cultures had a more stalk-like identity (Fig. 7B). Reflecting the methods of culture, the targeted UE cultures had nephron segments almost exclusively DN, while the organoid cultures also contained proximal segments (Fig. 7C). The absence of NPC-like cells in the DN-isolated and recultured cells while their presence in the directly differentiated UrEp may be explained by the different protocols used to generate UrEp and kidney developmental biology. Cultures differentiated towards an anteriorised intermediate mesoderm population directly from hPSCs are likely to generate a proportion of NPC-like cells as a bona fide posterior intermediate mesoderm of a more anterior nephrogenic cord, such as the mesonephric tubules. In contrast, the DN-derived cultures were depleted of mesenchymal cells.

When we compare the distribution of probabilities for the nephron and UrEp populations between samples, we see a broader range of scores in both targeted cultures compared to the Uchimura organoids (Fig. 7D, E). The targeted cultures are differentiated in the absence of any supporting stromal populations, instead the signalling factors required for specifying the cell identity are added to the media. While this also occurs in the Uchimura et al. [45] organoids, they are further supported by additional mesenchymal populations as would be the case in vivo*.* An emerging understanding of the importance of stroma in signalling and patterning both in vivo and in vitro may provide some key as to why there is less specificity within these direct, isolated cultures [83, 85].

### Gene expression database and interactive app

The capacity to investigate published single cell datasets is limited. The analysis provided in an original publication is generally the only output available without downloading and reanalysing a dataset. Kidney Interactive Transcriptomics (KIT) site (http://humphreyslab.com/SingleCell/) [86] provides analysis of a number of published HFK and organoid datasets [12, 20, 87] together with data from adult human and mouse healthy and injured kidney [86, 88, 89], allowing visualization of genes within these datasets in TSNE and Expression Plot formats. The Cello interactive app (https://cello.shinyapps.io/kidneycellexplorer/) [18] provides a visualization of gene expression broken down by carefully annotated subpopulations of the kidney nephron as expressed within adult mouse tissues. These useful tools enable the investigation of gene expression in kidney cell types; however, they do not provide a way to directly compare different datasets for relative proportions or levels of gene expression within component cells. We provide an interactive shiny app, freely available (https://kidneyregeneration.github.io/DevKidCC/articles/ShinyApp.html) [58], which allows for the investigation of gene expression in all organoid datasets as classified using *DevKidCC*. Using this App, gene expression can be directly compared between any included datasets, allowing for real-time investigation by all researchers. The tool provides a resource for researchers interested in gene expression and/or cell populations to identify the organoid protocol that would best suit their application, whether that be developmental biology, drug screening or clinical purposes.

## Discussion

The question of cell identity is one that is difficult to answer. Histologically, we can try to define a cell type based on its morphology, gene expression or protein expression, the latter typically being read by immunohistochemistry and immunofluorescence assays. In many cellular states, particularly those present during organogenesis, evaluation of cellular identity by functional assays is challenging and marker expression is rarely unique. This challenge is significant when evaluating cell identity using single-cell RNA sequencing data. Such data is sparse, providing an incomplete snapshot rather than a comprehensive picture. As capture technology and bioinformatics tools have improved, increased levels of information can be extracted from this data, providing an overall synergy of expression profile for groups of cells within a sample. This can be combined with the pseudotime trajectory or even molecular lineage tagging to relate cells within a sample by history, assisting in likely classification of cell type. Such inferences are much more difficult in a synthetic in vitro system such as hPSC-derived organoids. Such protocols direct cells to undergo a series of changes that attempt to replicate the in vivo process. However, in reality hPSC-derived lineages often do not completely recapitulate their in vivo counterparts, at least at the level of the transcriptome. We can often identify a gene, or a number of genes, expressed in a cell that provides information of its identity, but in many cases, there is ambiguity. This is compounded by our knowledge that hPSC-derived organoid models replicate early developmental cell states that are frequently in flux, not present in adult tissue and are less well defined.

The classification of cells within all single-cell data has been inconsistent as clustering and classification decisions vary between individual researchers and the limitations within each dataset. The arbitrary nature of classifying cells using clustering algorithms is challenged when identifying cells transitioning between populations, often represented as the ‘borders’ of clusters. The cluster-based classification of such cells will change with different approaches to analysis. The application of a cell-centred identification approach circumvents this challenge. *DevKidCC* represents a method of specifically classifying individual cellular identity within hPSC-derived kidney organoids based predominantly upon set models trained on a comprehensive reference dataset. It should be noted however that these models are indirectly dependant on cluster-based analysis as the reference itself was initially annotated this way. Our tool facilitates direct comparisons between kidney organoid datasets by classifying cells based on the reference data. The base package, *scPred* [53], includes a way to integrate the data within the models using *Harmony* [56], although this can introduce false correlations and over-corrections between similar cell populations such as the mesenchymal cells that have intermediate to high scores for both stroma and NPC. However, batch differences are a confounding source of variation that must be taken into account. Hence, *DevKidCC* runs one round of harmonization using the *scPred’s* inbuilt application of *Harmony.* This leads to potential iPSC-derived off-target populations with muscle or neural gene expression to be classified as NPC. This may be a result of the binary classifier for NPC not having enough information to delineate the cell types that have diverged from the NPC developmental trajectory, an example of in vitro artefacts. NPCs are an interesting population as some key markers, including *CITED1*, are not actually required for NPCs to become nephrons but are involved in other regulatory processes. To incorporate biological knowledge to refine the NPC classification, *DevKidCC* performs a further evaluation of *PAX2* expression to refine NPC classification. *PAX2* is a known in vivo marker of nephron identity not expressed in the stroma. Indeed it has been shown to repress stromal identity [60] and is an accurate marker of nephron lineage identity in single-cell kidney datasets. The classification for all datasets has been integrated into functions allowing for plotting any novel dataset in direct comparison using the classification from *DevKidCC*. A suite of custom visualisation functions is included in *DevKidCC* to provide a classification and visualization toolset to investigate cell identity and gene expression within novel and existing kidney organoids.

*DevKidCC* was developed so that it could be applied to novel datasets facilitating direct comparisons to those previously generated. This will make comparative studies much easier, facilitating the analysis of genetic variants, disease states or methodological variation in new protocols. While this system has developed a model with three tiers of subclassification, the complexity of the human nephron, even in the fetal kidney, is such that there is scope to interrogate individual cellular identity even further within this and other subcomponents. As these models were trained using developing HFK, the ability of the tool to accurately classify cell identity during earlier stages of mesoderm patterning or mature kidney is limited. The adult kidney shows significant specification of functional cell types within all segments of the final nephron, many of which have distinct functional roles in renal filtration and fluid homeostasis but are not present in the fetal organ. Indeed, the ratio of epithelium to stroma is dramatically shifted in the adult. While the fetal kidney begins to show expression of maturing cellular states, including expression of intercalated and principal cell identities within the distal nephron/collecting duct, it is likely that a distinct cellular identity tool will be required for the accurate identification of cellular identity in postnatal kidney tissue. Conversely, the use of HFK from Trimester 1 and 2 as the reference dataset limits the ability to identify earlier stages of morphogenesis. This may explain the large percentage of unassigned cell calls in datasets in early stages of kidney organoid differentiation protocols (Fig. 4A). However, *DevKidCC* applied to early-stage differentiations (day 7, intermediate mesoderm) split cell identity between NPC and unassigned, suggesting that the tool is able to identify those cells beginning to commit to the mesenchymal precursors of the kidney. Indeed, in a dataset that includes day 7, 15 and 29 organoids between two cell lines [14], there is a direct relationship between the proportion of cells classified as NPC at day 7 to the proportion of nephron cells at day 15 and 29 (Fig. 5B). We conclude that at this early stage the cells identified as NPC at this early stage could be the percentage of the differentiation correctly patterned to intermediate mesoderm and are still the cells that will go on to form the nephron population.

The generation of mature nephron structures is a challenge still facing the field and is a focus of current research. It is generally accepted that current organoid protocols generate tissues transcriptionally and morphologically similar to trimester 1 and 2 stages of development. To fully utilise organoids for disease and toxicology studies, optimisation of protocols to generate mature and functionally relevant tissues is essential. Here we show how the addition of retinoic acid impacts the cellular composition of organoids by depleting NPC cells when added after the point at which nephrogenesis has begun at day 12 in Takasato organoids. This also seems to increase the percentage of classifiable renal stroma compared to off-target mesenchyme, as well as increasing the proportion of EPT compared to other nephron sub-types. While there was minimal transcriptional evidence for an increase in podocyte maturation, the improved localisation of PEC marker CLDN1 to cells surrounding the podocytes in the glomerulus would indicate there is a positive effect on glomerular maturation.

## Conclusions

*DevKidCC* provides a robust, reproducible and computationally efficient tool for the classification of kidney single-cell data, in both human and organoid-derived tissue. Using *DevKidCC*, we can now directly compare between kidney samples regardless of batch and have done so for all available published datasets. This important advance has provided insights into differences in organoids derived using different protocols and allows for any novel dataset to be directly compared to all previous datasets. The included custom functions simplify visualisation of cell identity proportion and gene expression within samples and between multiple samples. Any novel dataset can be classified using the framework provided in this package, allowing for direct comparison to all previous datasets, all of which are included within the package. For visualisation of gene expression profiles and organoid cell identities, the gene expression profiles of all datasets have been built into an *R* Shiny app available at https://kidneyregeneration.github.io/DevKidCC/articles/ShinyApp.html [58] that does not require the use of *R* directly, allowing for easy access to this information. Finally, while this package has been built using HFK data to classify kidney cells, the framework can be transferred to any tissue type where adequate single-cell data is available.

## Supplementary Information

**Additional file 1. **This file contains **Figures S1** to **S4**.

**Additional file 2. **This file contains **Table S1A-B**.

**Additional file 3. **This file contains **Table S2**.

## Data Availability

*DevKidCC* is available from Github at https://github.com/KidneyRegeneration/DevKidCC [57]. *DevKidCC Kidney Organoid Gene Expression* interactive shiny dashboard is available at https://sbwilson91.shinyapps.io/devkidcc_interactive/ [58] and from Github at https://github.com/KidneyRegeneration/DevKidCC_Interactive [58]. This software is freely available to use, copy, modify, merge, publish and distribute subject to copyright under the MIT Licence (https://github.com/KidneyRegeneration/DevKidCC/blob/main/LICENSE). Single-cell RNA-sequencing human fetal kidney datasets can be found in GEO (GSE102596, GSE114530) and EMBL-EBI ArrayExpress (E-MTAB-9083) [21, 23, 28]. Single-cell RNA-sequencing organoid datasets can be found in GEO (GSE118184, GSE109718, GSE119561, GSE114802, GSE115986, GSE132026, GSE124472, GSE152014, GSE161255, GSE152685, GSE131086) [25, 30, 33–35, 40, 42, 44, 46, 48]. The single-cell RNA-sequencing organoid dataset generated in this study is available at GSE165408 [90].

## Abbreviations

HFK: Human fetal kidney
UTip: Ureteric tip
UOS: Ureteric outer stalk
UIS: Ureteric inner stalk
SPC: Stromal progenitor cells
CS: Cortical stroma
MS: Medullary stroma
MesS: Mesangial cells
Endo: Endothelium
NPC: Nephron progenitor cell
EN: Early nephron
EDT: Early distal tubule
DT: Distal tubule
LOH: Loop of Henle
EPT: Early proximal tubule
PT: Proximal tubule
PEC: Parietal epithelial cell
EPod: Early podocytes
Pod: Podocytes
UrEp: Ureteric epithelium
DN: Distal nephron
PN: Proximal nephron
RC: Renal corpuscle
AUROC: Area under the receiver operating characteristic curve
AURPG: Area under the precision recall gain curve
SEM: Standard error of the mean
ES: Embryonic stem cell
iPS: Induced-pluripotent stem cell
hPSC: Human-derived pluripotent stem cell
MET: Mesenchyme-to-epithelial transition
RA: Retinoic acid
KIT: Kidney Interactive Transcriptomics
TSNE: t-Distributed stochastic neighbour embedding
UMAP: Uniform manifest approximation and projection
