# Supplementary material for: DevKidCC allows for robust classification and direct comparisons of kidney organoid datasets
Source: Genome Med. 2022 Feb 22;14:19. doi: 10.1186/s13073-022-01023-z (PMC8862535; doi:10.1186/s13073-022-01023-z)
Supplement: Supplementary file 1 — Additional file 1. This file contains Figures S1 to S4. [file 13073_2022_1023_MOESM1_ESM.pdf]

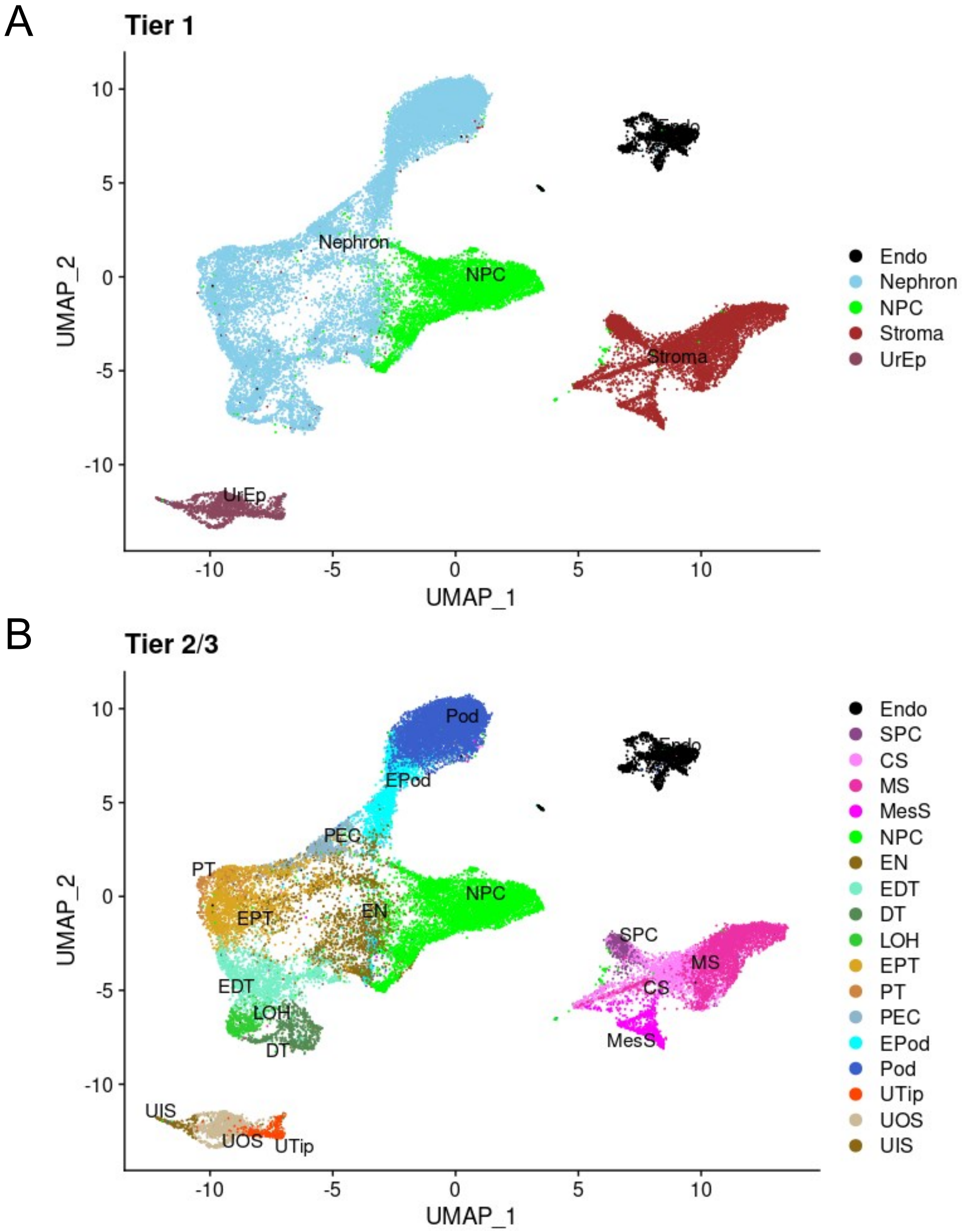

Fig S1: Tier 2 and 3 annotation of human fetal kidney reference

A) UMAP visualisation of the integrated reference HFK datasets annotated by tier 1 identities.

B) UMAP visualisation of the integrated reference HFK datasets annotated by tier 2/3 identities. HFK; human fetal kidney.

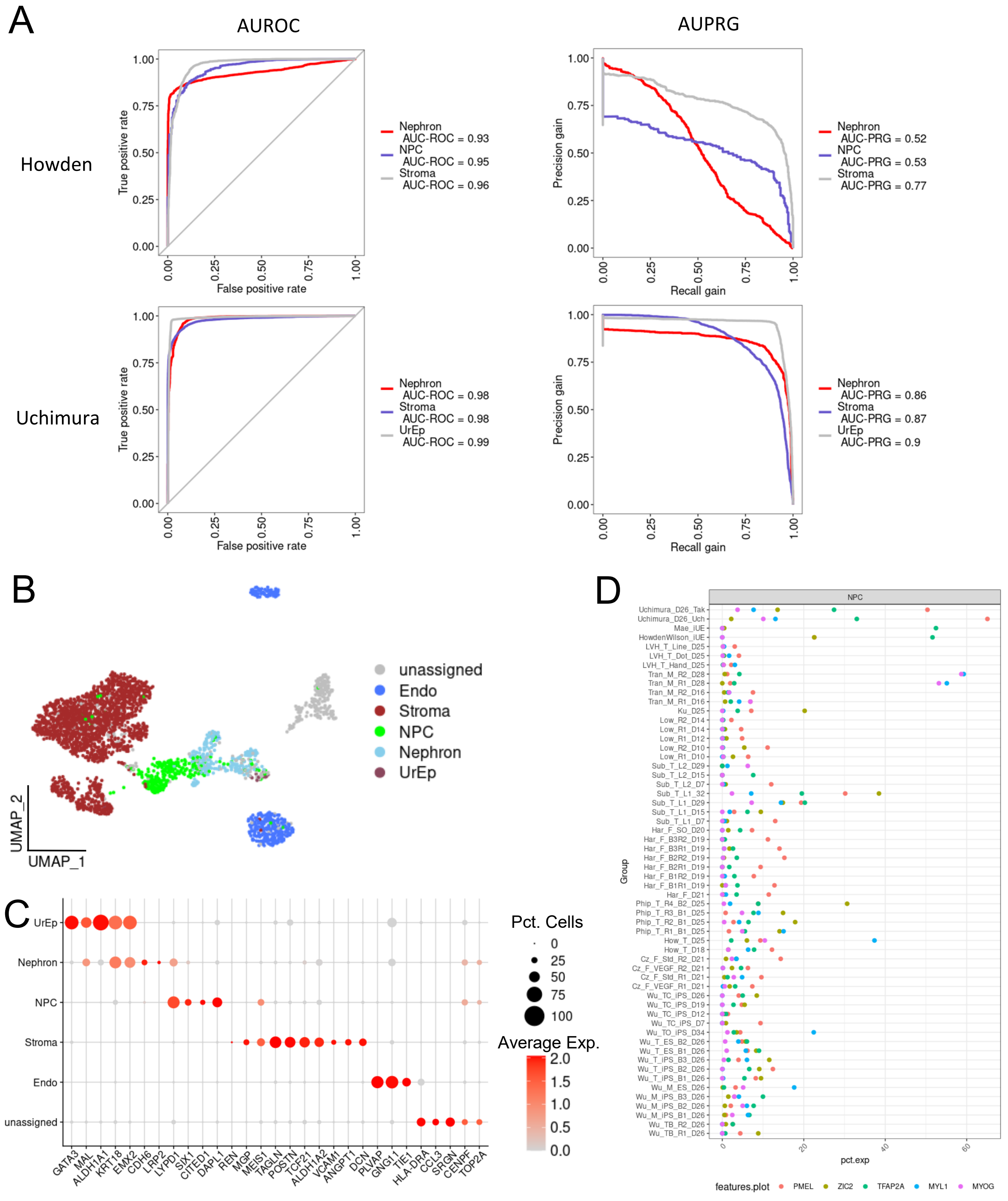
A
AUROC
AUPRG
Howden
Uchimura
True positive rate
False positive rate
Precision gain
Recall gain
0.00
0.25
0.50
0.75
1.00
Nephron AUC-ROC = 0.93
NPC AUC-ROC = 0.95
Stroma AUC-ROC = 0.96
Nephron AUC-PRG = 0.52
NPC AUC-PRG = 0.53
Stroma AUC-PRG = 0.77
Nephron AUC-ROC = 0.98
Stroma AUC-ROC = 0.98
UrEp AUC-ROC = 0.99
Nephron AUC-PRG = 0.86
Stroma AUC-PRG = 0.87
UrEp AUC-PRG = 0.9
B
unassigned
Endo
Stroma
NPC
Nephron
UrEp
UMAP_2
UMAP_1
C
UrEp
Nephron
NPC
Stroma
Endo
unassigned
GATA3 MAL ALDH1A1 KRT18 EMX2 CDH6 LRP2 LYPD1 SIX1 CITED1 DAPL1 REN MGP MEIS1 TAGLN POSTN TCF21 ALDH1A2 VCAM1 ANGPT1 DCN PLVAP GNG11 TIE1 HLA-DRA CCL3 SRGN CENPF TOP2A
Pct. Cells
0
25
50
75
100
Average Exp.
2.0
1.5
1.0
0.5
0.0
D
NPC
Group
Uchimura_D26_Tak
Uchimura_D26_Uch
Mae_iUE
HowdenWilson_iUE
LVH_T_Line_D25
LVH_T_Dot_D25
LVH_T_Hand_D25
Tran_M_R2_D28
Tran_M_R1_D28
Tran_M_R2_D16
Tran_M_R1_D16
Ku_D25
Low_R2_D14
Low_R1_D14
Low_R1_D12
Low_R2_D10
Low_R1_D10
Sub_T_L2_D29
Sub_T_L2_D15
Sub_T_L2_D7
Sub_T_L1_32
Sub_T_L1_D29
Sub_T_L1_D15
Sub_T_L1_D7
Har_F_SO_D20
Har_F_B3R2_D19
Har_F_B3R1_D19
Har_F_B2R2_D19
Har_F_B2R1_D19
Har_F_B1R2_D19
Har_F_B1R1_D19
Har_F_D21
Phip_T_R4_B2_D25
Phip_T_R3_B1_D25
Phip_T_R2_B1_D25
Phip_T_R1_B1_D25
How_T_D25
How_T_D18
Cz_F_Std_R2_D21
Cz_F_VEGF_R2_D21
Cz_F_Std_R1_D21
Cz_F_VEGF_R1_D21
Wu_TC_iPS_D26
Wu_TC_iPS_D19
Wu_TC_iPS_D12
Wu_TC_iPS_D7
Wu_TO_iPS_D34
Wu_T_ES_B2_D26
Wu_T_ES_B1_D26
Wu_T_iPS_B3_D26
Wu_T_iPS_B2_D26
Wu_T_iPS_B1_D26
Wu_M_ES_D26
Wu_M_iPS_B3_D26
Wu_M_iPS_B2_D26
Wu_M_iPS_B1_D26
Wu_TB_R2_D26
Wu_TB_R1_D26
0
20
40
60
pct.exp
features.plot PMEL ZIC2 TFAP2A MYL1 MYOG

Figure S2: Classifier performance evaluation on organoid samples

A) AUROC and AURPG metrics of binary classifiers as measured on two annotated organoid samples. B) UMAP visualisation of the Tran week 15 HFK dataset grouped by the tier 1 level of classification using DevKidCC. C Expression of marker genes used in Lindstrom to identify cell lineages. D) Percentage of cells per organoid sample expressing notable off-target genes, in the NPC classified population. HFK; human fetal kidney, NPC; Nephron Progenitor Cells.

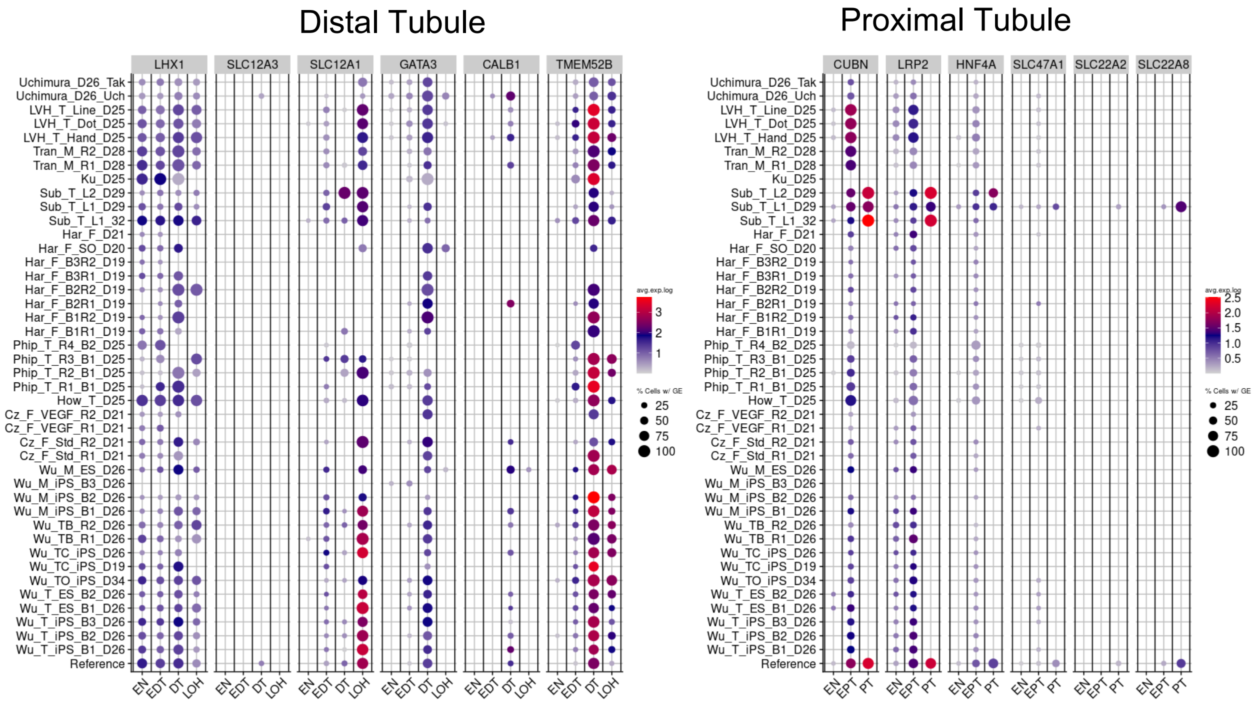

Figure S3: Expression of proximal and distal markers in organoids

A) Expression of key distal markers *LHX1*, *SLC12A3*, *SLC12A1*, *GATA3*, *CALB1* and *TMEM52B* in distal nephron segments across organoids. B) Expression of key proximal markers *CUBN*, *LRP2*, *HNF4A*, *SLC47A1*, *SLC22A2* and *SLC22A8* in proximal nephron segments across organoids

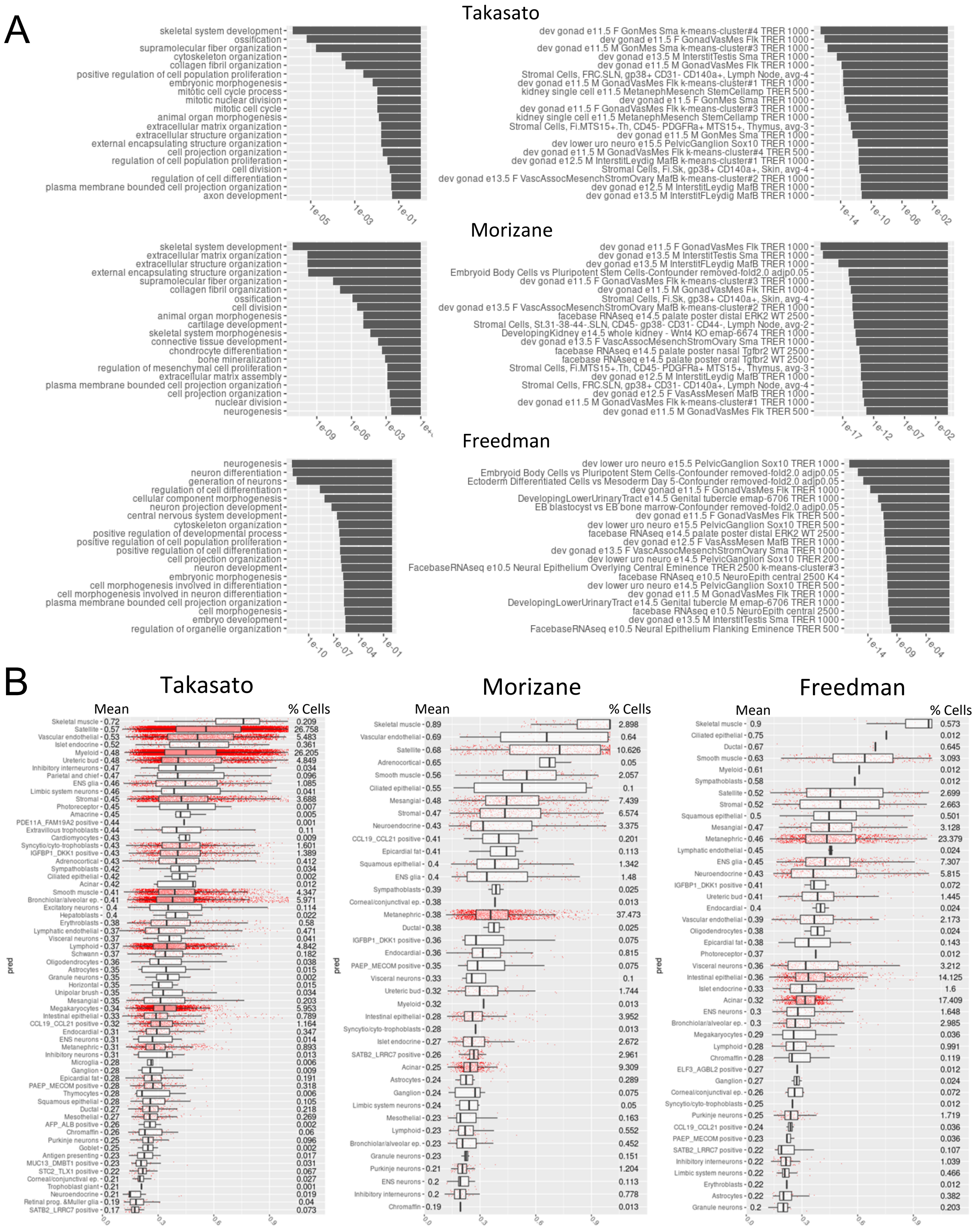

A
Takasato
skeletal system development
ossification
supramolecular fiber organization
cytoskeleton organization
collagen fibril organization
positive regulation of cell population proliferation
embryonic morphogenesis
mitotic cell cycle process
mitotic nuclear division
mitotic cell cycle
animal organ morphogenesis
extracellular matrix organization
extracellular structure organization
external encapsulating structure organization
cell projection organization
regulation of cell population proliferation
cell division
regulation of cell differentiation
plasma membrane bounded cell projection organization
axon development
dev gonad e11.5 F GonMes Sma k-means-cluster#4 TRER 1000
dev gonad e11.5 F GonadVasMes Flk TRER 1000
dev gonad e11.5 M GonMes Sma k-means-cluster#3 TRER 1000
dev gonad e13.5 M InterstitTestis Sma TRER 1000
dev gonad e11.5 M GonadVasMes Flk TRER 1000
Stromal Cells, FRC.SLN, gp38+ CD31- CD140a+, Lymph Node, avg-4
dev gonad e11.5 M GonadVasMes Flk k-means-cluster#1 TRER 1000
kidney single cell e11.5 MetanephMesench StemCellamp TRER 500
dev gonad e11.5 F GonMes Sma TRER 1000
dev gonad e11.5 F GonadVasMes Flk k-means-cluster#3 TRER 1000
kidney single cell e11.5 MetanephMesench StemCellamp TRER 1000
Stromal Cells, Fi.MTS15+.Th, CD45- PDGFRa+ MTS15+, Thymus, avg-3
dev gonad e11.5 M GonMes Sma TRER 1000
dev lower uro neuro e15.5 PelvicGanglion Sox10 TRER 1000
dev gonad e11.5 M GonadVasMes Flk k-means-cluster#4 TRER 500
dev gonad e12.5 M InterstitLeydig MafB k-means-cluster#1 TRER 1000
Stromal Cells, Fi.Sk, gp38+ CD140a+, Skin, avg-4
dev gonad e13.5 F VascAssocMesenchStromOvary MafB k-means-cluster#2 TRER 1000
dev gonad e12.5 M InterstitLeydig MafB TRER 1000
dev gonad e13.5 M InterstitFLeydig MafB TRER 1000
1e-05
1e-03
1e-01
1e-14
1e-10
1e-06
1e-02
Morizane
skeletal system development
extracellular matrix organization
extracellular structure organization
external encapsulating structure organization
supramolecular fiber organization
collagen fibril organization
ossification
cell division
animal organ morphogenesis
cartilage development
skeletal system morphogenesis
connective tissue development
chondrocyte differentiation
bone mineralization
regulation of mesenchymal cell proliferation
extracellular matrix assembly
plasma membrane bounded cell projection organization
cell projection organization
nuclear division
neurogenesis
dev gonad e11.5 F GonadVasMes Flk TRER 1000
dev gonad e13.5 M InterstitTestis Sma TRER 1000
dev gonad e13.5 M InterstitFLeydig MafB TRER 1000
Embryoid Body Cells vs Pluripotent Stem Cells-Confounder removed-fold2.0 adjp0.05
dev gonad e11.5 F GonadVasMes Flk k-means-cluster#3 TRER 1000
dev gonad e11.5 M GonadVasMes Flk TRER 1000
Stromal Cells, Fi.Sk, gp38+ CD140a+, Skin, avg-4
dev gonad e13.5 F VascAssocMesenchStromOvary MafB k-means-cluster#2 TRER 1000
facebase RNAseq e14.5 palate poster distal ERK2 WT 2500
Stromal Cells, St.31-38-44-.SLN, CD45- gp38- CD31- CD44-, Lymph Node, avg-2
DevelopingKidney e14.5 whole kidney - Wnt4 KO emap-6674 TRER 1000
dev gonad e13.5 F VascAssocMesenchStromOvary Sma TRER 1000
facebase RNAseq e14.5 palate poster nasal Tgfbr2 WT 2500
facebase RNAseq e14.5 palate poster oral Tgfbr2 WT 2500
Stromal Cells, Fi.MTS15+.Th, CD45- PDGFRa+ MTS15+, Thymus, avg-3
dev gonad e12.5 M InterstitLeydig MafB TRER 1000
Stromal Cells, FRC.SLN, gp38+ CD31- CD140a+, Lymph Node, avg-4
dev gonad e12.5 F VasAssMesen MafB TRER 1000
dev gonad e11.5 M GonadVasMes Flk k-means-cluster#1 TRER 1000
dev gonad e11.5 M GonadVasMes Flk TRER 500
1e-09
1e-06
1e-03
1e-17
1e-12
1e-07
1e-02
Freedman
neurogenesis
neuron differentiation
generation of neurons
regulation of cell differentiation
cellular component morphogenesis
neuron projection development
central nervous system development
cytoskeleton organization
positive regulation of developmental process
positive regulation of cell population proliferation
positive regulation of cell differentiation
cell projection organization
neuron development
embryonic morphogenesis
cell morphogenesis involved in differentiation
cell morphogenesis involved in neuron differentiation
plasma membrane bounded cell projection organization
cell morphogenesis
embryo development
regulation of organelle organization
dev lower uro neuro e15.5 PelvicGanglion Sox10 TRER 1000
Embryoid Body Cells vs Pluripotent Stem Cells-Confounder removed-fold2.0 adjp0.05
Ectoderm Differentiated Cells vs Mesoderm Day 5-Confounder removed-fold2.0 adjp0.05
dev gonad e11.5 F GonadVasMes Flk TRER 1000
DevelopingLowerUrinaryTract e14.5 Genital tubercle emap-6706 TRER 1000
EB blastocyst vs EB bone marrow-Confounder removed-fold2.0 adjp0.05
dev gonad e11.5 F GonadVasMes Flk TRER 500
dev lower uro neuro e15.5 PelvicGanglion Sox10 TRER 500
facebase RNAseq e14.5 palate poster distal ERK2 WT 2500
dev gonad e12.5 F VasAssMesen MafB TRER 1000
dev gonad e13.5 F VascAssocMesenchStromOvary Sma TRER 1000
dev lower uro neuro e14.5 PelvicGanglion Sox10 TRER 200
FacebaseRNAseq e10.5 Neural Epithelium Overlying Central Eminence TRER 2500 k-means-cluster#3
facebase RNAseq e10.5 NeuroEpith central 2500 K4
dev lower uro neuro e14.5 PelvicGanglion Sox10 TRER 500
dev gonad e11.5 M GonadVasMes Flk TRER 1000
DevelopingLowerUrinaryTract e14.5 Genital tubercle M emap-6706 TRER 1000
facebase RNAseq e10.5 NeuroEpith central 2500
dev gonad e13.5 M InterstitTestis Sma TRER 1000
FacebaseRNAseq e10.5 Neural Epithelium Flanking Eminence TRER 500
1e-10
1e-07
1e-04
1e-01
1e-14
1e-09
1e-04
B
Takasato
Mean
% Cells
Morizane
Mean
% Cells
Freedman
Mean
% Cells
pred

Figure S4: Expression profiles and classification of unassigned cells

A) The DEGs for all samples grouped by protocol were analysed using ToppFun (https://toppgene.cchmc.org/enrichment.jsp) to identify GO terms for biological process (left column) and co-expression atlas (right column) terms. Values are the Bonferroni adjusted p-value. B) The unassigned cells grouped by protocol were analysed using Azimuth (https://azimuth.hubmapconsortium.org/) with the Human Fetal Development atlas. Cells were plotted by most probable cell type match within the reference, arranged from the highest probability mean to lowest. Average mean and total % of cells from the query datasets also presented. TRER; top-relative-expression-ranked
